# Stack of Bi_2_Se_3_/Carbon Films with Pyramid Interface for Dual-Mode Temperature–Pressure Sensing in Aquatic Environments

**DOI:** 10.1007/s40820-026-02254-0

**Published:** 2026-06-22

**Authors:** Yan Xu, Xuefei Zhang, Size Lou, Zhe Tang, Dongmei Xie, Chuanrui Zhang, Mengran Chen, Heng Liu, Chuan Sun, Yixiang Ou, Peng-an Zong

**Affiliations:** 1 Jiangsu Collaborative Innovation Center for Advanced Inorganic Function Composites, College of Materials Science and Engineering, Nanjing Tech University, Nanjing, 211816 People’s Republic of China; 2 State Key Laboratory of New Ceramics and Fine Processing, School of Materials Science and Engineering, Tsinghua University, Beijing, 100084 People’s Republic of China; 3 Advanced Institute for Materials Research (WPI-AIMR), Tohoku University, Sendai, 980-8577 Japan; 4 National Innovation Institute of Defense Technology, Academy of Military Sciences of the People’s Liberation Army of China, Beijing, 100850 People’s Republic of China; 5 Joint Laboratory of Advanced Energy Materials and Intelligent Equipment, Beijing Academy of Science and Technology, Beijing, 100000 People’s Republic of China

**Keywords:** Bi_2_Se_3_, Thermoelectric, Underwater, Dual-mode sensing, Electromagnetic interference shielding

## Abstract

With the growing demand for marine exploration and environmental monitoring, underwater sensing technology faces severe challenges due to high humidity and the need for simultaneous detection of multiple physical parameters, such as temperature and pressure. Traditional underwater sensors typically rely on combinations of various functional materials to achieve dual-mode temperature–pressure detection, often requiring additional waterproof encapsulation, which complicates system integration. Achieving dual-mode temperature–pressure detection within a single material underwater remains particularly challenging. Herein, a waterproof dual-mode sensor based on Bi_2_Se_3_/carbon paper (CP) composite films is developed by electrochemically depositing pyramid-interface-structured Bi_2_Se_3_ layers on both sides of a flexible CP substrate. The film exhibits enhanced thermoelectric properties, with a power factor of 106.0 μW m^−1^ K^−2^ (9 times that of CP). Sensors constructed from vertically stacked pyramid-interface-structured films enable high-precision detection of temperature and pressure underwater, featuring a temperature-sensing response time of 0.9 s and a pressure sensitivity of 0.94% kPa^−1^, while allowing synchronous decoupling of the two signals. Moreover, the sensor exhibits excellent hydrophobicity, with a contact angle of 143.7°, along with robust stability, overcoming key limitations for underwater applications. This advance in flexible underwater sensing technology offers a reliable strategy for underwater human–machine interaction and monitoring.

**Supplementary Information:**

The online version contains supplementary material available at 10.1007/s40820-026-02254-0.

## Highlights

- A pyramid-interface-structured Bi_2_Se_3_ layer was electrochemically constructed on flexible carbon paper, exhibiting excellent hydrophobicity with a contact angle of 143.7°.
- Dual-mode decoupling of temperature and pressure without cross-interference was achieved using a single material in both air and underwater environments.
- The sensor maintains stable signal output under prolonged underwater operation, mechanical bending, electromagnetic interference exposure, and even direct flame contact, demonstrating exceptional environmental resilience.

**Supplementary Information:**

The online version contains supplementary material available at 10.1007/s40820-026-02254-0.

## Graphical abstract

**Figure:**
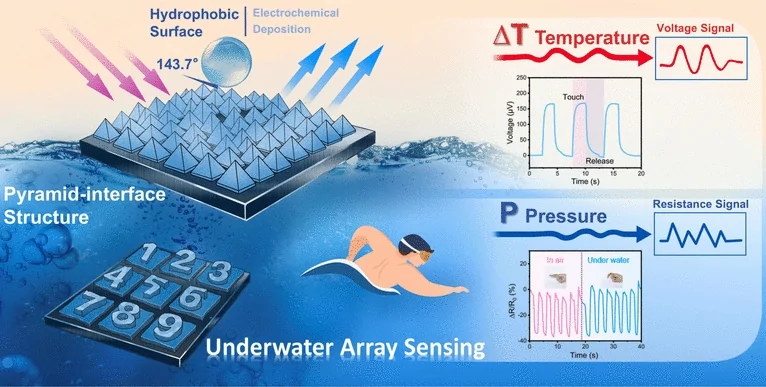

## Introduction

The growing demand for marine resource exploration and underwater environmental monitoring impose stringent requirements on sensing technology, particularly for the simultaneous and accurate detection of multiple physical quantity, such as temperature and pressure, under high-humidity conditions. Temperature fluctuations directly influence critical factors like dissolved oxygen levels and biological distribution, while pressure data are essential not only for depth positioning but also for early warnings of sea geological activity [1, 2]. Current underwater temperature sensing primarily relies on thermistor probes or fiber Bragg grating sensors [3, 4], whereas pressure detection commonly employs piezoresistive micro-electro-mechanical system (MEMS) sensors or titanium-alloy-encapsulated high-precision probes [5, 6]. To achieve dual‑parameter sensing in aqueous environments [7–9], sensors must integrate waterproof encapsulation with effective signal-decoupling capabilities. Conventional approaches often combine discrete temperature‑ and pressure‑sensing units through advanced circuit designs and packaging units [10, 11]. However, such integration increases structural complexity, raises the risk of signal interference, and complicates mass production. Furthermore, additional waterproof encapsulation layers further undermine system reliability and limit deployment in harsh underwater settings.

In contrast, dual-mode sensors based on a single sensing material can simultaneously respond to temperature and pressure, thereby simplifying device architecture and fabrication [12]. Early designs frequently employed heterogeneous composites, such as polydimethylsiloxane (PDMS)/carbon nanotube sponge (w-CNT) [13], laser-induced porous carbon/starch films [14], or laser-carburized graphene oxide/carboxymethylcellulose (LC-GO/CMC) [15], combining strain-sensitive materials with thermosensitive materials. However, these systems often suffer from interfacial delamination, and water infiltration can cause baseline-signal drift, reducing accuracy. To enhance water resistance, prior studies have introduced hydrophobic modification [16], applied separate hydrophobic coatings [17, 18], or employed protective encapsulation [19]. For instance, Ni et al. [20] deposited polypyrrole (PPy) on reduced graphene oxide (rGO)/polyurethane sponge followed by PDMS/n-hexane treatment, while Li et al. [21] modified cellulose/nanotube (CNF-CNT) fabric with octadecylamine and Zhu et al. [22] encapsulated electrochemically deposited Bi_2_Te_3_/carbon cloth with polyimide (PI). Such strategies typically rely on organic materials, such as PDMS, PPy, octadecylamine, PI, which involve complex processing, exhibit poor corrosion resistance over time and may swell or degrade upon prolonged water immersion. Moreover, such coatings often attenuate signal transmission and compromise comfort. Although inherently hydrophobic materials (e.g., fluorocarbon nanotubes, polyurethane materials) [23, 24] circumvent the need for extra layers, they typically cannot perform the integrated dual‑mode sensing of temperature and pressure required in aqueous environments.

Therefore, it is intriguing to design a single material that inherently combines bimode temperature–pressure sensing with excellent hydrophobicity for underwater sensing. Micro/nanoscale interface design offers a promising route to enhance hydrophobicity. Common approaches include constructing porous architectures (via chemical etching or templating) [25, 26] and biomimetic layered structures [27, 28] (e.g., lotus-leaf-like papillae). While these improve surface roughness and contact angles, porous networks may trap water or suffer mechanical stress failure in wet environments, limiting stability. In contrast, pyramid‑shaped structures exhibit superior hydrophobic performance in humid conditions due to their symmetrical gradient and apical curvature [29], which facilitate a stable Cassie–Baxter state [30], minimize solid–liquid contact, and promote droplet roll-off. Concurrently, this pyramid geometry induces a stress-concentration effect through its sloped facets [31], causing the contact area to vary nonlinearly with pressure, enabling high-pressure sensitivity across a broad range. Despite these advantages, the application of pyramid‑structured thermoelectric materials for dual‑mode temperature–pressure sensing has not yet been reported.

In this work, we fabricate a waterproof dual-mode sensor by electrodepositing continuous pyramid-structured Bi_2_Se_3_ on both sides of flexible carbon paper (CP) and forming a stacked Bi_2_Se_3_/CP/Bi_2_Se_3_ multilayer assembly. The pyramid interface endows the composite film with intrinsic hydrophobicity (contact angle ~ 143.7°). The resulting sensor demonstrates a wide detection range for both pressure and temperature, fast response (0.9 s), and long-term stability. It operates reliably in both dry and aquatic environments, tracking human motion as well as subtle underwater disturbances. This work thus provides a versatile platform for aquatic environmental sensing and human–machine interaction, advancing the development of wearable, amphibious dual‑mode sensors.

## Experimental Section

### Materials

This work employed pulsed electrodeposition to synthesize Bi_2_Se_3_ directly onto CP, which served as the working electrode (WE). The electrolyte contained 4 mM bismuth nitrate pentahydrate (Bi(NO_3_)_3_·5H_2_O, Aladdin, > 99%), 6 mM selenium dioxide (SeO_2_, Aladdin, > 99%), and 1 M nitric acid (HNO_3_, Nanjing Wanqing, 65.0%–68.0%). The abbreviations used in this work and their corresponding physical units are listed in Table S1.

### Synthesis of Bi_2_Se_3_/CP Film

A standard three‑electrode setup was used with platinum (Pt) as the counter electrode (CE) and saturated calomel electrode (SCE) as the reference electrode (RE). Deposition was carried out at room temperature without stirring, applying potentials of 0.00, − 0.02, − 0.04, and − 0.06 V. After deposition, the obtained Bi_2_Se_3_/CP film was rinsed thoroughly with deionized water, dried at 60 °C for 1.5 h, and then annealed at 300 °C for 30 min under a flowing argon atmosphere to enhance the crystallinity and electrical properties of the Bi_2_Se_3_ layer while minimizing the pronounced selenium volatilization and defect-related effects that may occur at higher temperatures [32, 33].

### Film Characterization

The samples were characterized by X-ray diffractometer (XRD; Rigaku, SmartLab) and field emission scanning electron microscope (FE-SEM, Phenom Pharos G1, Phenom-World) equipped with an energy-dispersive X-ray spectrometer (EDS). Elemental composition and chemical states were analyzed using X-ray photoelectron spectroscopy (XPS, Escalab 250 Xi, Thermo Scientific) with monochromatic 1486.6 eV Al K_α_ radiation at an incidence angle of 58°. Hall effect measurements were taken at room temperature under a magnetic field of 400 mT (CH-Magnetoelectric Technology, CH-100) to measure the carrier mobility (*μ*), carrier concentration (*n*), and conductivity (*σ*). The Seebeck coefficient (*S*) was measured using a portable Seebeck meter (JiaYitong Technology). Joule-heating performance was visualized with an infrared thermal camera (UNI-T, UTi 120 S). Electromagnetic shielding performance in the X-band (8.2–12.4 GHz) was measured with a vector network analyzer (VNA, Agilent, USA).

### Temperature and Pressure Sensor Testing

A sensor assembly was fabricated by physically stacking ten 1 cm × 2 cm Bi_2_Se_3_/CP films. Both ends of the stack were connected to silver wires using silver paste. To secure the electrical contacts and enhance robustness, the electrode termination areas on both sides of the device were encapsulated with a PI layer, while the central region remained exposed to either aqueous or atmospheric environments. The sensor was connected to a Keithley DMM6500 digital multimeter for synchronous real‑time data acquisition. The temperature-sensing signal was recorded as voltage fluctuations, while the pressure-sensing signal was characterized by the relative change in resistivity (Δ*R/R*_0_), calculated using Eq. (1):

1 $$\Delta R/{R}_{0}\left(\%\right) = \frac{R-{R}_{0}}{{R}_{0}}$$

where *R*_0_ and *R* are the resistance before and after pressure application. For underwater sensing, the sensor was fixed to the bottom of water‑filled container to ensure stable signal transmission. Therefore, the water depth used in the tests was ~ 10 cm, with a corresponding hydrostatic pressure of ~ 1 kPa, primarily focusing on experiments in shallow-water environments.

### Sensor Array

A 3 × 3 sensor array was constructed by vertically stacking Bi_2_Se_3_/CP thermoelectric films each unit. Each film measured 1 cm × 1 cm, with the number of stacked layers varying by position (2, 4, 6, 8, 10, 12, 3, 5, and 7 layers for positions 1–9, respectively). Electrically, each array cell was formed by connecting the left end of the uppermost film and the right end of the lowermost film with silver paste and silver wires, establishing an independent output path per unit. All top-layer silver wires were connected in parallel to a common top lead, and all bottom-layer wires to a common bottom lead, offering a uniform interface for the entire array and facilitating consistent signal monitoring across all sensing positions.

### Calculations

All density functional theory (DFT) calculations in this study were carried out using the Vienna Ab initio Simulation Package (VASP) [34]. Core electrons were described using projector-augmented wave (PAW) pseudopotentials, which are integrated within the VASP framework [35]. A plane-wave energy cutoff of 400 eV was adopted for the basis set [36]. Structural optimization was performed within the generalized gradient approximation (GGA) [37] employing the Perdew–Burke–Ernzerhof (PBE) exchange–correlation functional [38]. During relaxation, atomic positions were adjusted until the residual forces fell below 0.03 eV Å^−1^, and the total energy variation was constrained to within 10^−5^ eV. To probe the electronic structure, static calculations were conducted on a high-density k-point mesh with a spacing not exceeding 0.01 Å^−1^. The work function and charge density difference are analyzed via VASPKIT code [39].

Finite element modeling was performed to investigate the effect of film microstructure on stress distribution and contact area. In the geometric model, the initial distance between the upper and lower layers was kept constant. The material density, Young’s modulus, and Poisson’s ratio were set to 7700 kg m^−3^, 10^9^ Pa, and 0.1, respectively, which may differ from reality. Therefore, the simulated stress ratio was used for comparison instead of absolute value. The Solid Mechanics physics interface was used, and the material was defined as a linear elastic material. The lower layer was fixed, while a prescribed displacement was applied to the upper layer. The upper and lower layers were connected using a contact pair, and the augmented Lagrangian method was selected for contact modeling. A physics-controlled mesh was used, with the element size set to extra fine.

## Results and Discussion

### Synthesis, Phases, and Microstructures

**Fig. 1:**
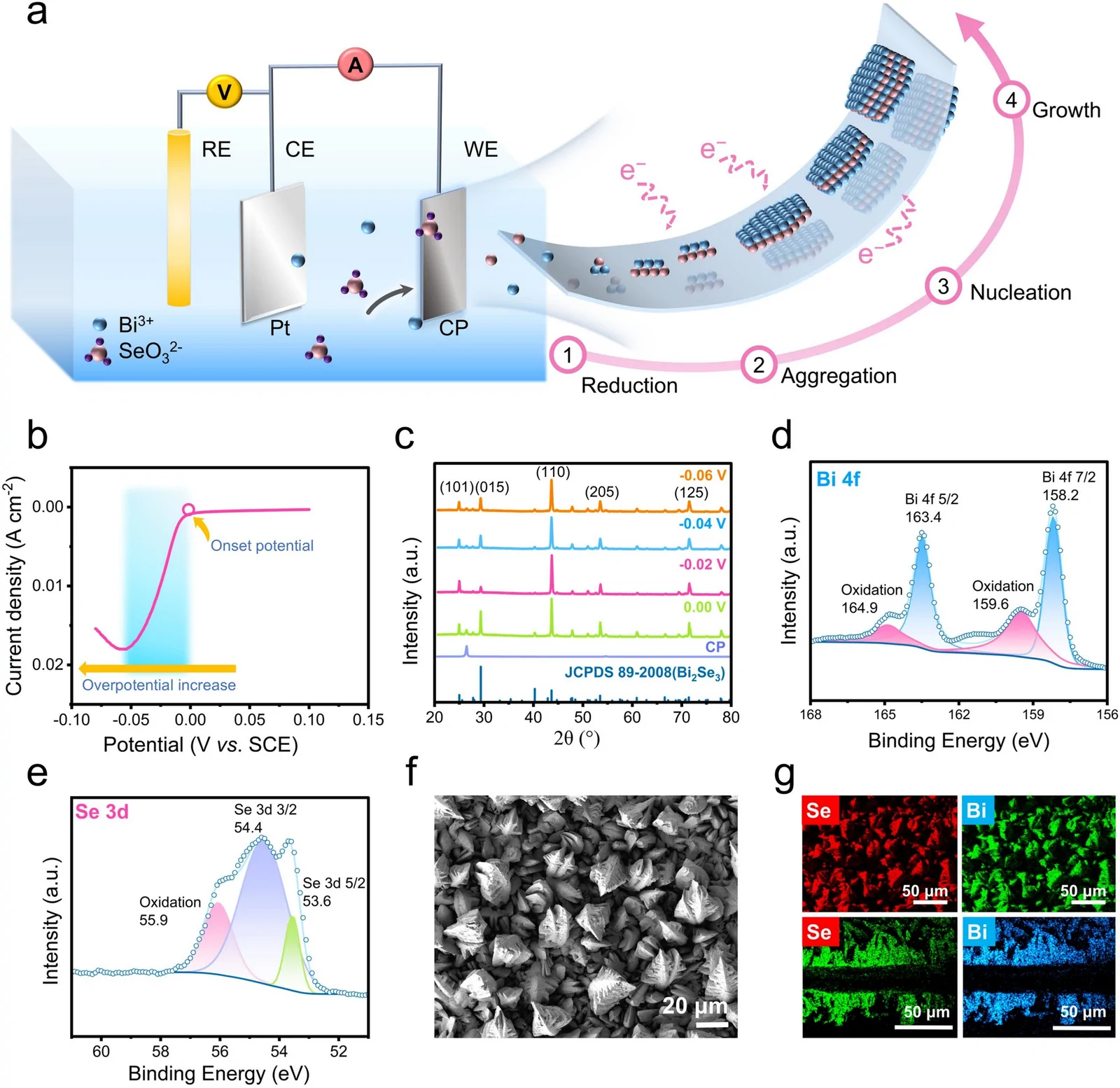
Synthesis and microstructure characterization of Bi_2_Se_3_/CP films: **a** Schematic of the electrodeposition synthesis process, including the sequential stages of reduction, ion aggregation, nucleation, and growth stages. **b** LSV curve recorded at a scan rate of 0.01 V s^−1^. prior to deposition. **c** XRD patterns of Bi_2_Se_3_/CP films deposited at different potentials. **d** High-resolution XPS spectra of the Bi 4*f* and **e** Se 3*d* region. **f** SEM image showing pyramid-like structure of the Bi_2_Se_3_/CP film. **g** EDS elemental mapping of the film surface (upper) and cross-sectional area (bottom)

The fabrication of Bi_2_Se_3_/CP film is illustrated in Fig. 1a, proceeding through four sequential stages: electrochemical reduction, ion aggregation, nucleation, and crystal growth. Prior to deposition, the CP substrate, ~ 21 μm thick, was rinsed with ethanol and deionized water to eliminate surface contaminants, thereby enhancing the interfacial adhesion of the subsequently deposited Bi_2_Se_3_. Electrodeposition under a pulsed deposition mode was performed in a standard three-electrode configuration, with the pretreated CP, Pt sheet, and SCE serving as WE, CE, and RE, respectively. The optimal deposition parameters were determined by linear sweep voltammetry (LSV) at a scan rate of 0.01 V s^−1^ prior to deposition, as shown in Fig. 1b. Under the applied electric field, Bi^3+^ and SeO_3_^2−^ ions migrate toward the CP substrate. Upon reaching the surface, they undergo electrochemical reduction and subsequent self-assembly, growing vertically to form a two-dimensional layered Bi_2_Se_3_ structure with the characteristic -Se-Bi-Se-Se-Bi-Se- quintuple-layer ordering [40]. The overall reaction can be represented by Eq. (2) [41]:

2 $$3{H}_{2}Se{O}_{3} + 2{Bi}^{3+} + 12{h}^{+} + 18{e}^{-} \to { Bi}_{2}{Se}_{3} + 9{H}_{2}O$$

The deposition potential, which directly regulates current density, plays a critical role in determining the composition and microstructure of the film. To systematically investigate this effect, deposition was performed at four distinct potentials: 0.00, − 0.02, − 0.04, and − 0.06 V. The XRD pattern of the Bi_2_Se_3_/CP film is shown in Fig. 1c. Aside from the peak at 26.4° corresponding to the CP substrate, all other diffractions match the rhombohedral phase of Bi_2_Se_3_ (JCPDS#89–2008), with characteristic reflections at 24.3°, 29.57°, and 43.6°. After annealing at 300 °C [33], a strong preferential orientation of the (110) crystal plane emerges, indicating predominant crystal growth along the *c*-axis perpendicular to the substrate. As the deposition potential shifts from 0.00 to − 0.06 V, the intensity of the (110) peak increases, reaching a maximum at − 0.02 V, and then decreases. Conversely, the intensity of the (105) peak follows an opposite trend, minimizing at − 0.02 V. This anomalous orientation competition phenomenon can be attributed to the dual effect of electrochemical polarization on crystal-growth kinetics. At the moderate reduction potential of − 0.02 V, the diffusion-layer thickness of Se^2+^ ions reaches an optimum, favoring vertical growth. At more negative potentials, intensified concentration polarization promotes lateral growth, causing the lower-surface-energy (015) crystal plane to become dominant again. Consequently, deposition at − 0.02 V yields the strongest (110) orientation and the highest overall crystallinity. Such pronounced (110) texture is known to enhance electron transport in Bi_2_Se_3_-based alloy [42, 43]. The promoted ion-ordered deposition at this potential likely results in a highly oriented, low-defect structure, which is expected to enhance *μ* and thereby optimizing the *S* and *σ*, a relationship that will be further discussed in the following section.

The chemical states of elements in the composite film were analyzed by XPS. As shown in Fig. 1d, e, the Bi 4*f* and Se 3*d* core‑level spectra of Bi_2_Se_3_/CP exhibit characteristic peaks along with oxide-related signals, indicating partial surface oxidation. In the Bi 4*f* spectrum (Fig. 1d), the doublet at 158.2 eV (Bi 4*f*_7/2_) and 163.4 eV (Bi 4*f*_5/2_) [44] is assigned to Bi in Bi_2_Se_3_, while the peaks at 159.6 and 164.9 eV correspond to Bi^3+^ in Bi_2_O_3_. Similarly, the Se 3*d* spectrum (Fig. 1e) shows peaks at 53.6 eV (Se 3*d*_5/2_) and 54.4 eV (Se 3*d*_3/2_) from Se in Bi_2_Se_3_ [45], accompanied by a higher‑binding‑energy component at 55.9 eV attributed to Se oxides. These results confirm the successful synthesis of Bi_2_Se_3_ and the surface oxidation of both Bi and Se elements. Figure 1f presents detailed SEM images of Bi_2_Se_3_/CP films electrodeposited at − 0.02 V. A uniform and dense coating of Bi_2_Se_3_ is observed on the CP substrate, composed of interlocked plate-like crystals that exhibit a pyramid-like morphology. EDS mapping **(**Figs. 1g and S1) further reveals a homogeneous distribution of Bi and Se elements across the film surface, consistent with the uniform microstructure seen in SEM.

To elucidate the formation of the pyramid-like microstructure and the effect of deposition amount on interfacial morphology, the surface evolution of Bi_2_Se_3_/CP films prepared with different deposition charges (10, 30, 50, and 70 C) at − 0.02 V was examined (Fig. S2). With increasing deposition charge, the surface morphology exhibits a clear stage-by-stage transition. At 10 C, the surface shows poor crystallinity, consisting mainly of rice-like particles and amorphous fine crystals. At 30 C, distinct crystalline protrusions and sheet-like crystals emerge. At 50 C, a well-defined, uniform pyramid-like microstructure with good crystallinity is formed. At 70 C, excessive deposition covers and fills the pyramids, resulting in a relatively dense clustered structure. These results indicate that the deposition charge critically regulates the interfacial configuration and roughness of the Bi_2_Se_3_/CP film, with 50 C identified as the optimal condition.

### Thermoelectric Performance

**Fig. 2:**
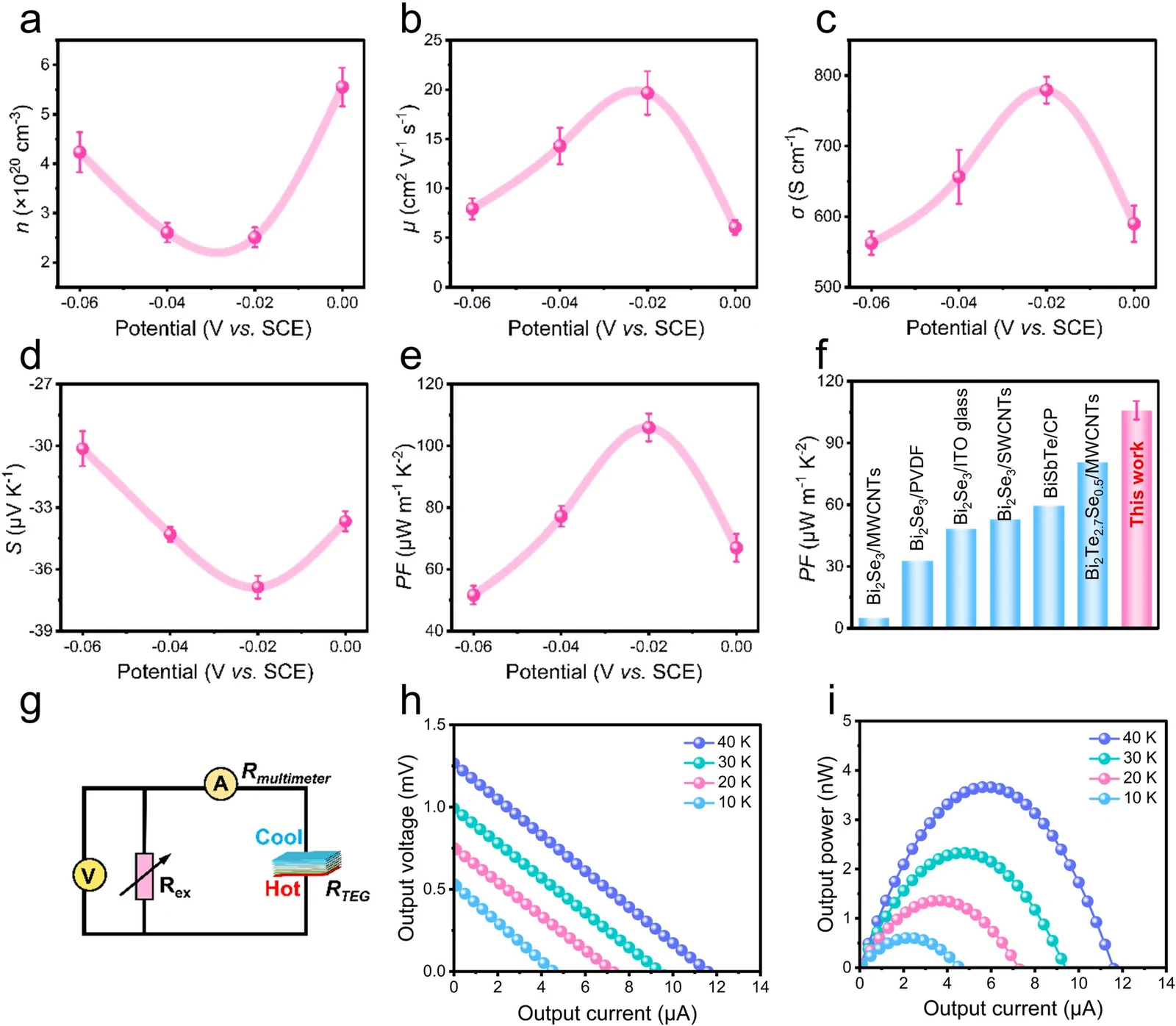
Thermoelectric properties of single-layer Bi_2_Se_3_/CP films and output performance of 10-layer stacked devices: **a** Carrier concentration *n*. **b** Carrier mobility *μ*. **c** Electrical conductivity *σ*. **d** Seebeck coefficient *S*, and **e** power factor PF of Bi_2_Se_3_/CP films deposited at different potentials. **f** Comparison of the maximum *PF* achieved in this work with previously reported. **g** Circuit diagram for measuring the output power of the thermoelectric device. **h, i** Output voltage and power as functions of current under temperature differences of 10, 20, 30, and 40 K

The relationship between deposition potential and the thermoelectric properties of the composite film is summarized in Fig. 2a–e and Table S2. As shown in Fig. 2a, the *n* initially decreases as the deposition potential shifts negatively from 0.00 to − 0.02 V. This trend arises because Se possesses a more positive reduction potential than Bi, leading to its preferential reduction at mildly negative potentials. At − 0.02 V, Bi and Se begin to co-reduce nearly stoichiometrically (~ 2:3), reducing vacancy and anti-site defect concentrations. When the potential is further shifted negatively from − 0.02 to − 0.06 V, *n* increases gradually, which can be attributed to the enhanced reduction of Bi, resulting in its overdeposition and the formation of cation vacancies ${V}_{Bi}^{\bullet \bullet }$. Figure 2b shows that the *μ* increases as the deposition potential shifts negatively from 0.00 to − 0.02 V. This improvement is due to the moderate negative potential promotes ordered, *c-*axis-oriented growth of Bi_2_Se_3_, forming a continuous layered structure that minimizes lattice defects and grain-boundary scattering. Further negative shifts intensify concentration polarization, increase grain-boundary density, and induce cation vacancies due to non‑stoichiometric Bi excess, collectively enhancing scattering, reducing *μ*. The *σ*, calculated as *σ* = *enμ*, is presented in Fig. 2c. Its variation closely follows that of *μ*, reaching a maximum of 779.3 S cm^−1^ at − 0.02 V where *μ* peaks. Theoretical calculations (Fig. S3a, b) indicate an intrinsic band gap of 0.44 eV for Bi_2_Se_3_. The near-zero gap predicted by DFT for the Bi_2_Se_3_/CP interface likely stems from modeling an idealized, perfect contact. Moreover, the work function (*Φ*) of Bi_2_Se_3_ decreases from 4.23 to 3.71 eV in Bi_2_Se_3_/CP (Fig. S3c, d), suggesting interfacial charge transfer that raises the Fermi level and leads to the DFT-predicted metallic state [46].

The *S* values of the Bi_2_Se_3_/CP samples are shown in Fig. 2d. According to Eq. (3) [47]:

3 $$\left|S\right| = \frac{{8\pi }^{2}{k}_{B}^{2}}{{3eh}^{2}}{m}^{*}T{(\frac{\pi }{3n})}^\frac{2}{3}$$

where *h* is Planck constant, *k*_B_ is Boltzmann constant, m^*^ is the effective mass of charge carriers, and *e* is the elementary charge, *S* is inversely proportional to *n*. Thus, as *n* increases, *S* gradually decreases, and vice versa. While pristine CP exhibits a low *S* value of 6.9 μV K^−1^, the Bi_2_Se_3_/CP film deposited at − 0.02 V achieves a significantly enhanced *S* of − 36.9 μV K^−1^, representing a fivefold increase over CP, with the negative sign confirming n‑type conduction.

Owing to the simultaneous increase in *S* and *σ*, the *PF* of Bi_2_Se_3_/CP reaches a maximum of 106.0 μW m^−1^ K^−2^ at − 0.02 V (Fig. 2e), representing a ninefold improvement over bare CP (11.9 μW m^−1^ K^−2^). Comparing with previously reported power factors of the Bi_2_Se_3_-based composites near room temperature, the present material demonstrates a clear advantage (Fig. 2f) [12, 48–52]. The pulsed electrodeposition employed here offers a key benefit over constant-potential electrodeposition: During the ON phase, metal ions are deposited, while their concentration at the electrode surface gradually decreases; during the OFF phase, ions are replenished within the diffusion layer. This cyclic process facilitates the formation of a uniform and dense Bi_2_Se_3_ layer, thereby enhancing the *PF* of the composite film.

The power output performance of the thermoelectric generator (TEG) fabricated by the Bi_2_Se_3_/CP film legs was evaluated by connecting it to a series of external resistors under varying temperature gradients (Δ*T*). A schematic of the measurement circuit is shown in Fig. 2g. As shown in Fig. 2h, the output voltage increases linearly from 0.5 to 1.3 mV as Δ*T* rises from 10 to 40 K. The corresponding output power, *P*, is calculated using Eq. (4) [53]:

4 $$P = \frac{{E^{2} }}{{\left( {\frac{{{\mathrm{R}}_{{{\mathrm{ex}}}} - R_{{{\mathrm{total}}}} }}{{{\mathrm{R}}_{{{\mathrm{ex}}}} }}} \right)^{2} + 4R_{{{\mathrm{total}}}} }}$$

where *E* is the output voltage of TEG under ∆*T*, *R*_ex_ and *R*_total_ represent the external resistance and internal resistance, respectively. When Δ*T* is fixed, the maximum power (*P*_max_) is achieved when *R*_ex_ = *R*_total_. Figure 2i illustrates the dependence of output power on output current: *P*_max_ increases from 0.6 to 3.7 nW as Δ*T* rises from 10 to 40 K.

### Sensor Fabrication and Temperature Sensing in Air and Underwater

Based on the Seebeck effect, the Bi_2_Se_3_/CP film can spontaneously generate a voltage signal in response to a temperature gradient, serving as the sensing element in a temperature sensor that requires no external power for its core transduction function. A device was fabricated by vertically stacking ten Bi_2_Se_3_/CP films, with electrical contacts established using silver paste and silver wires at both ends which sealed with PI, while the middle area is exposed out (Fig. S4). Comparative experiments on stacked devices with different layer numbers (8, 10, and 12 layers) were performed to evaluate both their temperature- and pressure-sensing performances. The output voltage increases with more layers due to enhanced carrier accumulation driven by Seebeck effect, but the response time also becomes longer (Fig. S5).

**Fig. 3:**
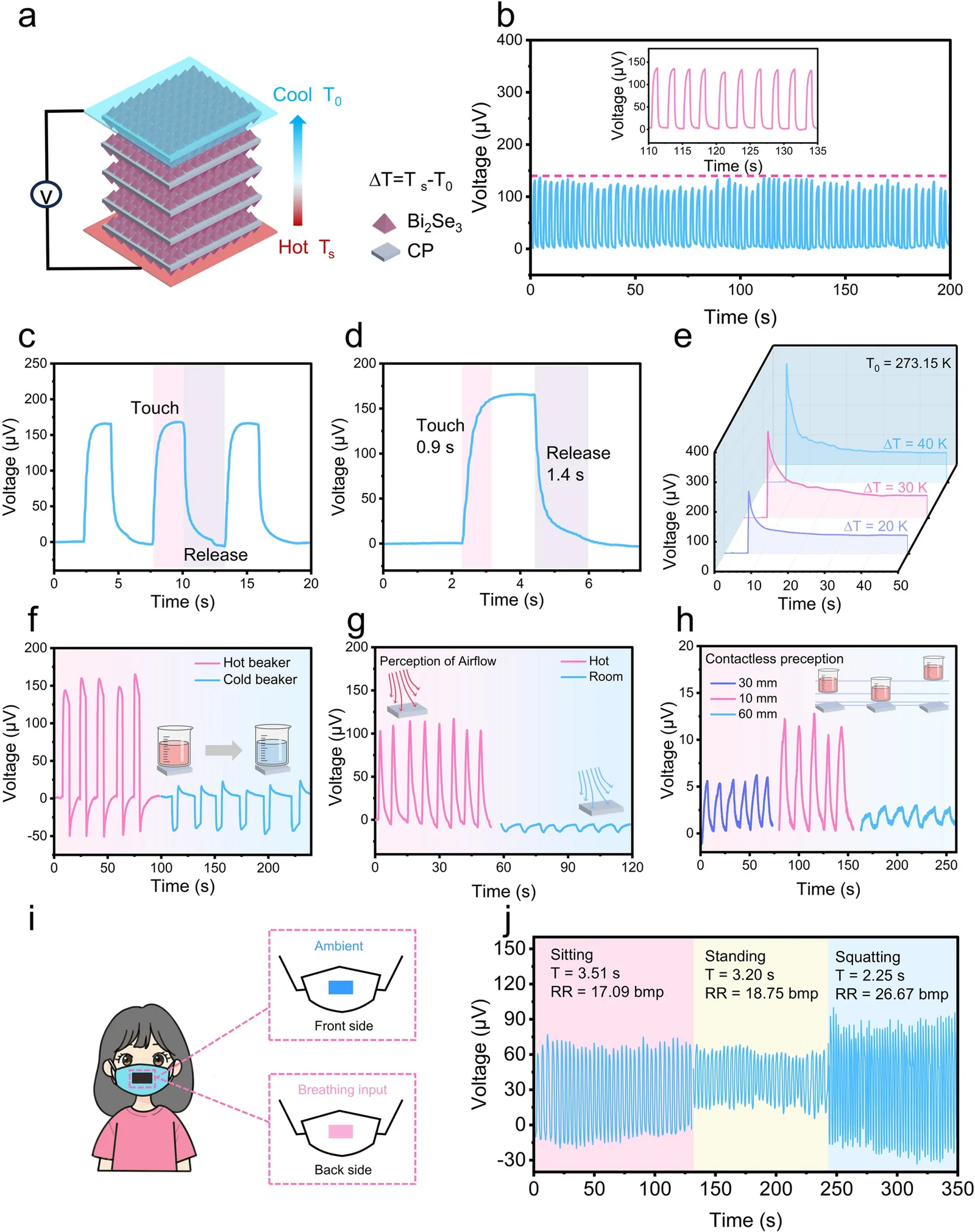
Structure design and temperature-sensing performance of the Bi_2_Se_3_/CP stacked sensor: **a** Schematic illustration of the sensor architecture (a 5-layer stack is shown for clarity; the actual device uses a 10-layer stack). **b** Voltage response under continuous finger contact for 200 s, demonstrating underwater operational stability. **c** Response and recovery behavior during repeated finger-touch-and-release cycles. **d** Single-finger-touch voltage output of the self-powered sensor, showing a response of 165 μV with a response time of 0.9 s and recovery time of 1.4 s. **e** Voltage response under applied temperature gradients ranging from 20 to 40 K. **f** Bidirectional thermoelectric response: positive voltage upon hot‑load application and negative voltage upon cold‑load application. **g** Real‑time voltage signals in response to cold and hot air flows. **h** Non-contact sensing performance: output voltage as a function of detection distance. **i** Integration of the sensor into a smart mask for respiratory monitoring; the rear side contacts the exhaled air (hot side), while the front side is exposed to ambient air (cold side). **j** Respiratory monitoring under different physiological states (standing, sitting, and rapid squatting), with respiratory rate (RR) and respiratory cycle (T) indicated

A schematic of the simplified device structure is depicted in Fig. 3a, highlighting the pyramid-shaped interface contacts between adjacent films. The underwater stability of the sensor was evaluated through a prolonged finger-touch test lasting 200 s, during which the output voltage remained steady (Fig. 3b). Compared to the continuous touch test conducted in water (Fig. S6), the sensor delivered a slightly higher output voltage underwater. This increase is attributed to the larger temperature difference between the finger and the device in the aqueous environment. Both tests confirm that the sensor maintains excellent operational stability in either environment. Figure 3c shows the voltage response to repeated touch-and-release cycles. A single touch produced an output voltage of 165 μV with a rapid response time of 0.9 s and a recovery time of 1.4 s (Fig. 3d), demonstrating rapid response and recovery underwater.

To assess temperature sensitivity, the sensor was affixed to the outer side of a beaker containing an ice–water mixture and then immersed in hot water of different temperatures (Figs. 3e and S7a). Upon immersion, the output voltage exhibited an instantaneous peak, followed by a gradual decay to a stable value. This transient behavior arises from the initial maximum Δ*T* between the inner and outer surfaces of the sensor: The inner side remains at the beaker wall temperature (~ 0 °C initially), while the outer side rapidly equilibrates with the surrounding hot water (Fig. S7b). As the beaker wall warms over time, the Δ*T* diminishes, causing the voltage to decrease until a steady state is reached (Fig. S7c). The steady-state output voltage increased consistently with applied Δ*T*, confirming its fine temperature resolution underwater. Moreover, the sensor exhibited clear bidirectional response: placement of a hot-water beaker induced a positive voltage, whereas an ice-water breaker generated a negative signal (Fig. 3f). Similarly, exposure to warm or cool air streams immediately produced corresponding positive or negative voltage signals (Fig. 3g). The device also exhibited effective non-contact sensing capability. Within a distance range of 10–60 mm, the output voltage varied inversely with detection distance (Fig. 3h), allowing spatial mapping of temperature gradients.

As shown in Fig. S8, the device generates a stable and distinguishable voltage output at a minimum temperature difference of Δ*T* = 0.2 K and maintains good response stability up to Δ*T* = 80 K, demonstrating a wide detection range. Figure S9 presents the relationship between Δ*T* and output voltage. The output voltage increases gradually with Δ*T*, exhibiting a good linear trend. Linear fitting yields a temperature sensitivity of *S*_T_ = 9.8 μV K^−1^. These results demonstrate that the device responds stably to small temperature differences and maintains reliable thermoelectric output over a wide Δ*T* range, confirming its good sensitivity and applicability for temperature detection. Comparison with previously reported underwater or dual-mode sensors reveals that the proposed sensor achieves a competitive overall performance (Table S3). Unlike most existing dual-mode sensors that are not suitable for underwater operation, this device functions reliably in underwater environments. In terms of dynamic response, the sensor exhibits a pressure response time of 0.2 s and a temperature response time of 0.95 s, which are comparable or superior to many reported systems (e.g., porous TPU: 0.19 s for pressure but lacks temperature sensing). Furthermore, while the temperature sensitivity is lower than some non-underwater devices, the device offers a unique combination of underwater operation capability, dual-parameter sensing, and balanced sensitivity–response characteristics.

To illustrate a practical application for temperature sensing in wet air, the sensor was integrated into a smart mask for real-time respiratory monitoring based on the temperature difference between exhaled and inhaled airflow (Fig. 3i). Positioned near the nose and mouth, the sensor is exposed both to respiratory wet airflow and ambient air. It reliably tracked respiratory rate (RR), a key physiological parameter for metabolic assessment and early clinical warning [54]. Systematic evaluation revealed distinct RR under different activity states (Fig. 3j): 17.09 breaths per minute (bpm) (cycle time, *T* = 3.51 s) while sitting, 18.75 bpm (*T* = 3.20 s) when standing, and 26.67 bpm (*T* = 2.25 s) during rapid breathing. Notably, the signal amplitude increased significantly during squatting, indicating heightened respiratory intensity.

### Pressure Sensing in Air and Underwater

**Fig. 4:**
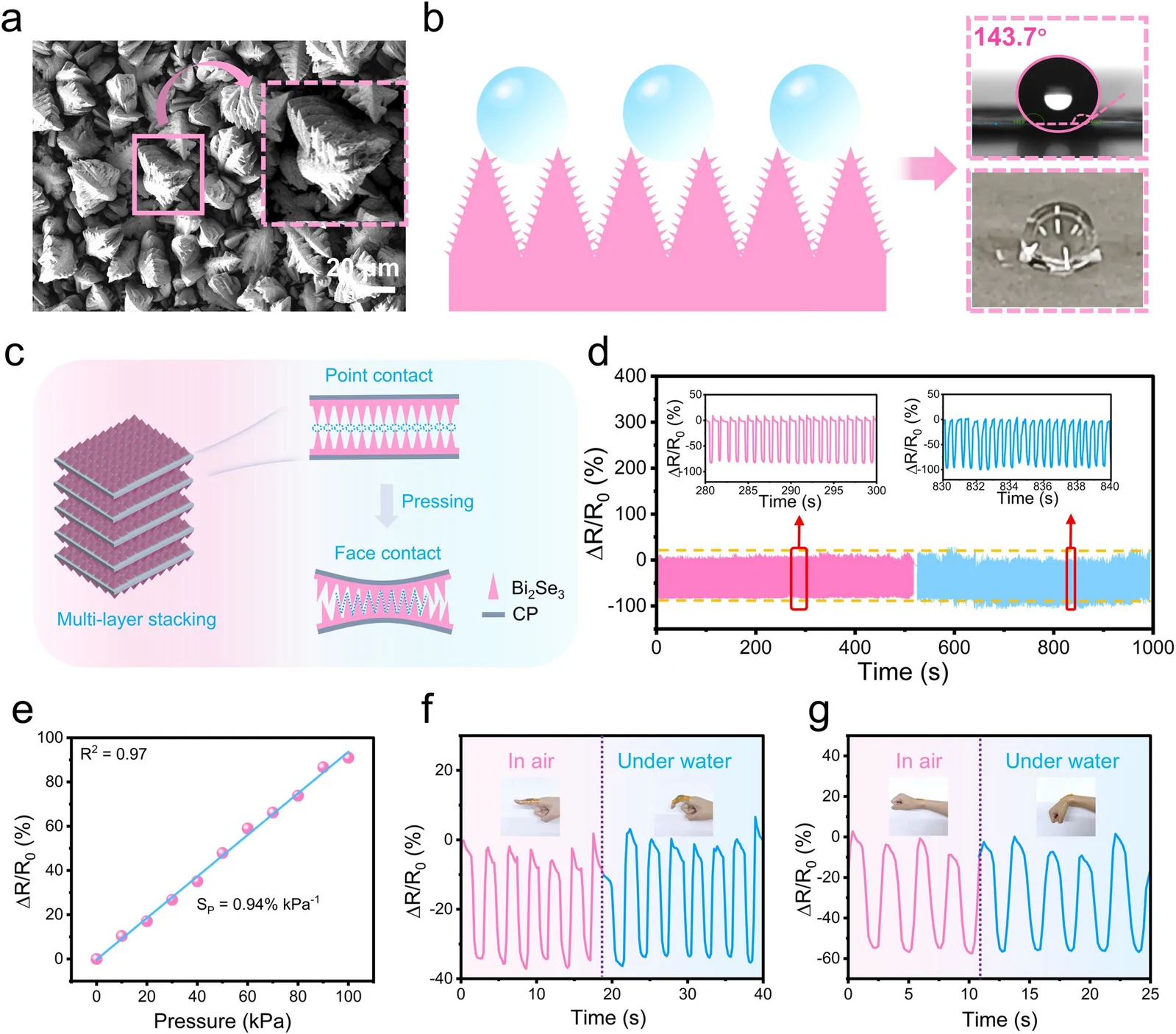
Pressure-sensing performance of the Bi_2_Se_3_/CP composite film in air and underwater: **a** SEM image of the film surface; inset: magnified view showing the pyramid-like microstructure. **b** Hydrophobicity characterization: schematic of the solid–air–liquid interface, water contact angle measurement, and optical photograph. **c** Operating principle of the pressure sensor: schematic illustration of resistance variation under applied pressure due to increased interlayer contact area. **d** Relative resistance change (Δ*R/R*_0_) over 1000 s during cyclic compression-release tests in air and underwater, demonstrating excellent stability and repeatability. **e** Resistance change ratio versus Pressure curve. Real‑time resistance signals in response to human joint motions: **f** finger bending and **g** wrist bending

As shown in Fig. 4a, a surface SEM image of the Bi_2_Se_3_/CP film is presented, with the inset revealing a magnified view. The surface exhibits a distinctive fractal morphology consisting of pyramid-like microstructures with nanoscale edge roughness. This hierarchical architecture facilitates air entrapment and establishes a stable solid–air–liquid composite interface, leading to pronounced hydrophobicity. Figure 4b illustrates this hydrophobic mechanism, along with a contact-angle test schematic and a photographic inset, confirming a water contact angle of up to 143.7° that approaches superhydrophobicity. To confirm that the remarkable hydrophobicity originates from surface microstructure regulation, the water contact angles of Bi_2_Se_3_/CP films prepared with different deposition charges under − 0.02 V were measured. As shown in Fig. S10a–d, the contact angles for 10, 30, 50, and 70 C are 99°, 120.3°, 143.1°, and 111.8°, respectively. The 50 C sample exhibits the largest contact angle, indicating the best hydrophobicity. Combined with the morphological evolution, these results suggest that a moderately developed, uniformly distributed pyramid-like microstructure is most favorable for constructing a rough hydrophobic interface, enhancing air trapping and stabilizing the solid–air–liquid composite contact state. Insufficient deposition leads to inadequate surface roughness, while excessive deposition fills the pyramids and densifies the surface, both detrimental to optimal hydrophobicity. Thus, the excellent hydrophobicity is closely linked to the pyramid-like hierarchical structure, and the 50 C morphology represents an optimal balance between roughness and structural uniformity.

As shown in Fig. 4c, the pyramid-stacked structure endows the sensor with exceptional pressure sensitivity, as the progressive increase in interlayer contact area systematically expands the conductive network. To further reveal this interfacial effect, the underwater pressure responses of stacked devices assembled from samples prepared with different deposition charges (10, 30, 50, and 70 C) were compared (Fig. S11a–d). The relative resistance changes are − 63%, − 89%, − 97%, and − 77%, with corresponding response/recovery times of 1.1/1.0, 0.5/0.4, 0.12/0.18, and 0.4/0.8 s, respectively. The device based on the 50 C sample exhibits the largest resistance change and the fastest response/recovery, indicating the best pressure-sensing performance. These results demonstrate that the pyramid-like interfacial structure facilitates interlayer contact reconstruction and conductive pathway regulation, whereas overly smooth or overly dense surfaces are unfavorable for optimal pressure response.

As shown in Figs. S12 and S13, the pyramid-stacked structure exhibits a gradual increase in effective contact area (*A*) and uniform stress distribution under compression (0–100% displacement), whereas the island-stacked structure shows contact saturation within 0–40% displacement and severe local stress concentration, e.g., at 20% and 40% displacement, the relative stress in the island structure is 23.4 and 14.5 times that of the pyramid structure, respectively. These differences directly affect device performance. First, according to the contact resistance (*R*_c_) relation, *R*_c_ ∝ 1/*A*, the larger and more progressive increase in contact area of the pyramid structure leads to a greater reduction in *R*_c_ under compression, thereby enabling higher pressure sensitivity and larger resistance modulation. Second, the lower and more uniformly distributed stress in the pyramid structure avoids localized stress accumulation, reducing the risk of mechanical damage and performance degradation, while also extending the effective displacement range for pressure monitoring. These simulation results are consistent with our experimental observations and with previous finite-element studies [55].

As shown in Fig. S14, the resistance-change amplitude and response time also vary considerably with layer number. Notably, a larger number of layers do not simply lead to better performance; instead, there is an optimal balance between signal enhancement and interfacial contact regulation. Among the three configurations, the 10-layer device achieves the best overall balance in terms of both output magnitude and response speed for dual-mode sensing. Therefore, it was selected as the representative structure for this study. Stability tests conducted in both air and underwater environments demonstrate excellent repeatability over 1000 s of cyclic finger pressure (Fig. 4d). To further evaluate the operational stability of the device under conditions closer to a realistic marine saline environment, an additional long-term stability test in a simulated seawater salt environment was conducted. Specifically, the device was first immersed in a 5 wt% NaCl solution at 60 ºC for 100 h, followed by a sensing stability test. As shown in Fig. S15, after the high-temperature saline immersion treatment, the device still maintained relatively stable output signals during a continuous 1000 s test, indicating good environmental tolerance and operational stability in the simulated seawater saline environment.

As shown in Fig. S16, the device achieves a minimum detectable pressure of 0.1 kPa and an upper test limit of 100 kPa. Within this range, the pressure–response curve (Fig. 4e) reveals a distinct linear sensitivity region, with a sensitivity of *S*_P_ = 0.94% kPa^−1^. The sensor precisely monitors human joint movements, such as finger bending, wrist rotation, and elbow flexion, through corresponding resistance changes (Figs. 4f, g and S17), highlighting its potential for wearable biomechanical sensing in diverse environments, including underwater sports or rehabilitation. Beyond limb motion, it also captures subtle facial expressions on moist skin. When attached to a moist face, distinct signals are generated during smiling, pouting blinking, and frowning (Fig. S18a–d), demonstrating its ability to detect microexpressions under conditions of sweat or water exposure, which is an essential feature for underwater communication and emotion-aware diving assistants. This high-precision detection provides valuable data for applications in post-exercise monitoring, psychological assessment, emotion recognition, across varied environments, particularly in aquatic and marine settings where conventional sensors often fail.

**Fig. 5:**
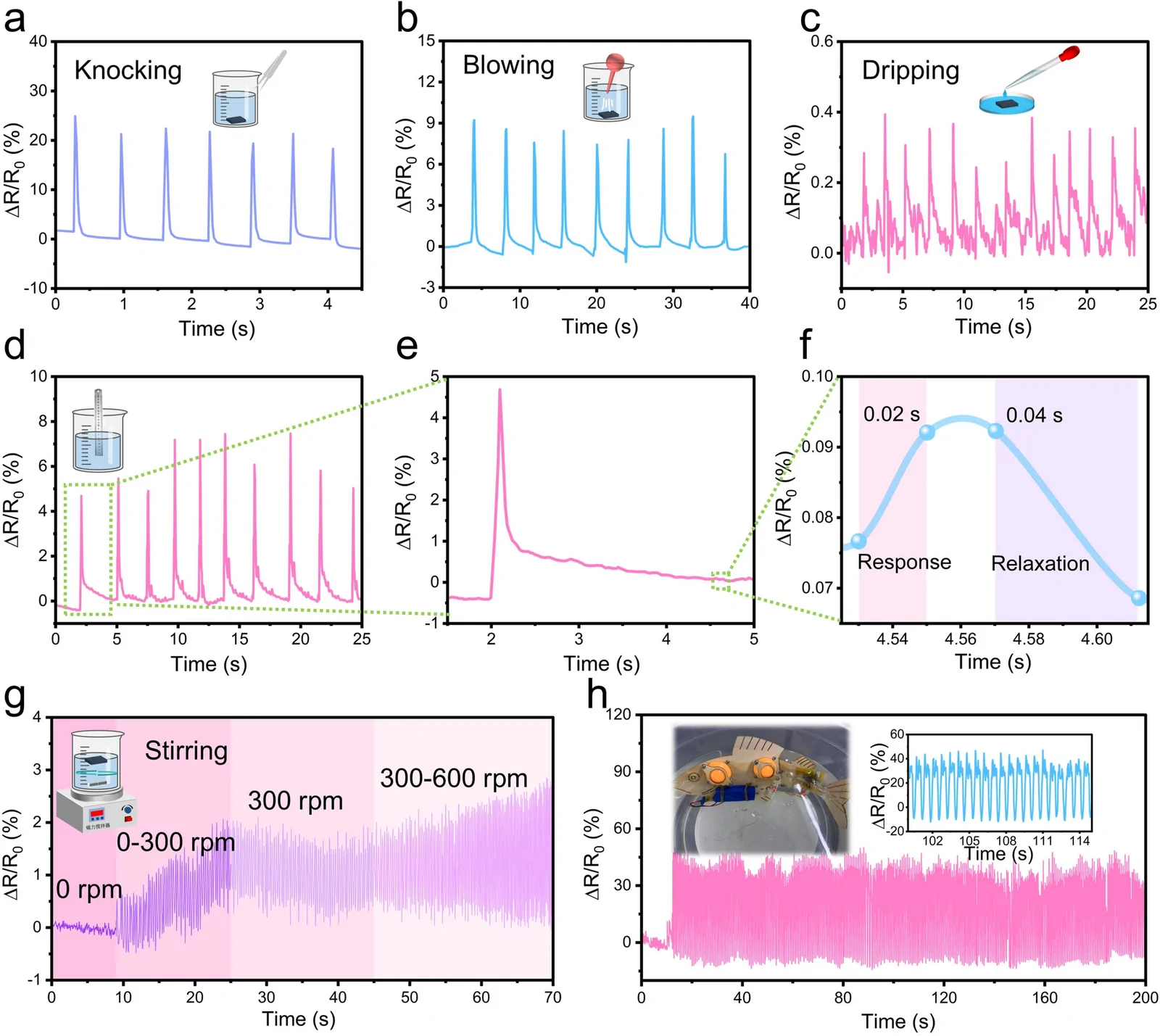
Underwater pressure-sensing performance under various dynamic disturbances: **a** Resistance response to light tapping on the outer wall of the water‑filled container. **b** Signal variation induced by periodic air puffs directed at the submerged sensor using a syringe. **c** Instantaneous resistive response triggered by a single water droplet falling onto the sensor surface. **d** Vibration tracking via a steel ruler partially submerged in water; the sensor is attached to the ruler to monitor damped oscillations. **e** Enlarged view of a single vibration cycle from **d**. **f** Response time and recovery time from the damped signal profile from **e**. **g** Real‑time resistance changes under controlled stirring speeds (0–600 rpm) in the aquatic environment. **h** Continuous monitoring of underwater motion for 200 s using a sensor-mounted on a robotic fish during tail‑flapping propulsion

The pressure-sensing performance of the device in underwater environments was evaluated through a series of simulated perturbations. The sensor was mounted inside a water-filled container to replicate submerged operation. As shown in Fig. 5a, light taps on the container wall with tweezers elicited clear transient resistance signals, which returned promptly to baseline after each stimulus. In Fig. 5b, periodic air puffs directed at the submerged sensor via a syringe induced slight but detectable resistance shifts, demonstrating potential for sensing subtle pressure variations near the water surface. The sensor exhibited high sensor sensitivity to minute stimulus, as evidenced by the instantaneous resistance response triggered by a single water droplet falling onto its submerged surface (Fig. 5c). To quantify dynamic response, the sensor was attached to a vibrating steel ruler. It reliably tracked damped oscillations across different frequencies and amplitudes (Fig. 5d–f) with a fast response time of 0.02 s and a recovery time of 0.04 s, sufficient for capturing human motion. Further tests examined the sensing behavior under hydrodynamic flow. Resistance changes were recorded while stirring the water at controlled rotational speeds (0–600 rpm), as shown in Fig. 5g. The results confirm its sensitivity to minor flow perturbations while maintaining the overall signal stability. To simulate sustained dynamic pressure in an aquatic setting, the sensor was mounted on a robotic fish undergoing tail-flapping motion for 200 s (Fig. 5h). Regular, periodic resistance variations were detected throughout the swimming cycle, underscoring the robustness and stability for long-term underwater motion tracking. In addition, the experimental results presented in this work were all obtained under shallow-water conditions. Its applicability and long-term operational stability in deep-sea high-pressure environments require further verification.

### Cross-talk Free Dual-Mode Detection in Air and Underwater

**Fig. 6:**
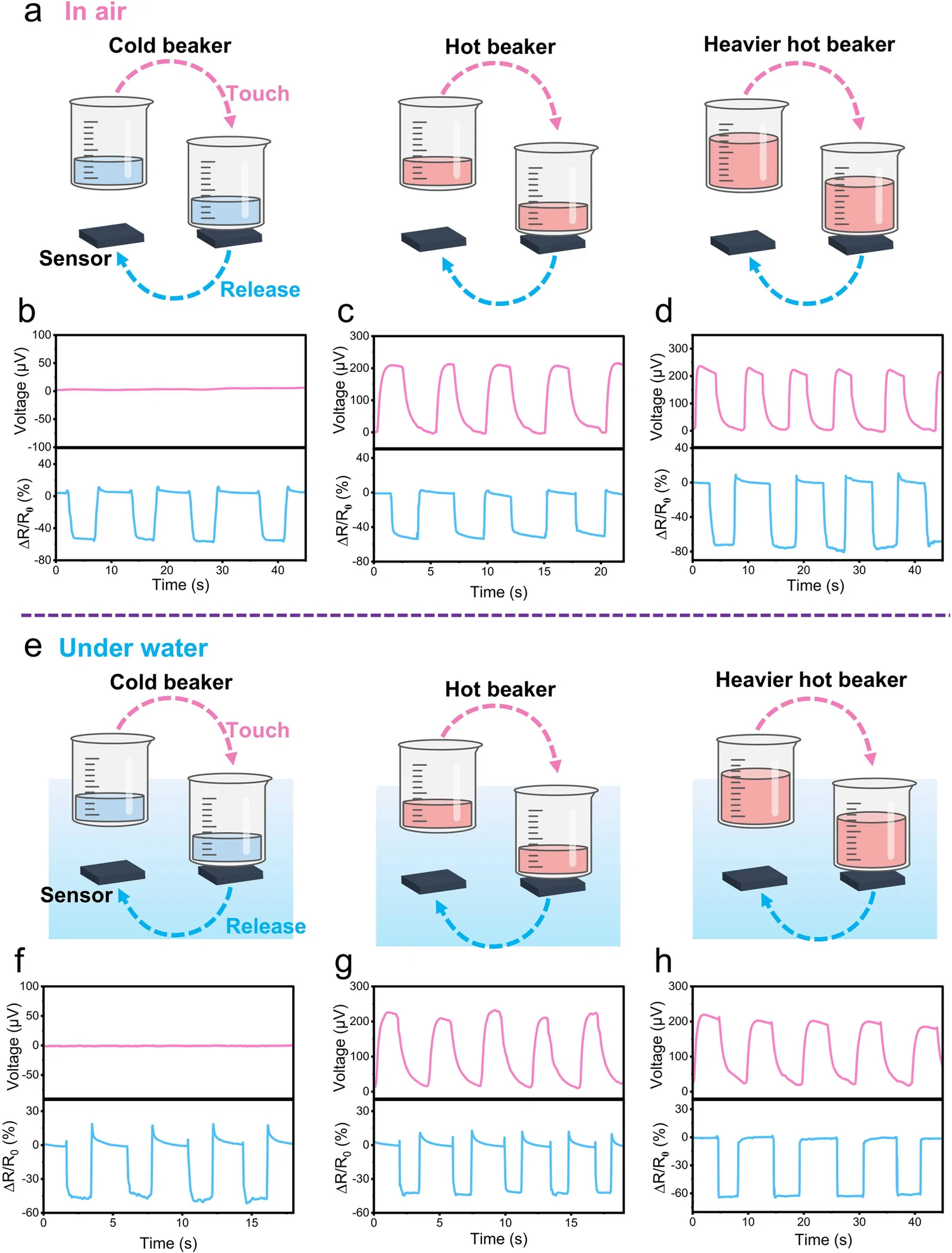
Dual‑mode temperature–pressure-sensing performance in air and underwater environments: **a** Schematic illustration of the experimental setup in air: beakers containing cold water, hot water, and a larger volume of hot water (heavier load) are sequentially placed on and removed from a 10-layer Bi_2_Se_3_/CP stacked sensor. **b–d** Corresponding real‑time voltage (temperature) and resistance (pressure) responses measured in air under the conditions shown in **a**. Both signals remain stable and exhibit no observable cross-interference. **e** Schematic of the equivalent experimental procedure conducted with the sensor fully submerged in water. **f–h** Voltage and resistance responses recorded underwater corresponding to the conditions in **e**. The output signals show that the obtained sensing signals remain essentially unchanged compared to those measured in air, demonstrating excellent stability

To evaluate the dual-parameter sensing performance, a controlled decoupling experiment was conducted (Fig. 6a). Beakers containing a small amount of cold water, a small amount of hot water, and a large amount of hot water were sequentially placed on a dual-mode sensor composed of a 10-layer Bi_2_Se_3_/CP stack. The output signals, a pink voltage curve (temperature response) and blue resistance curve (pressure response), exhibit distinct characteristics due to their different physical origins. In air, placing the cold-water beaker induced only a ~ 45% pressure-related resistance change. As the water temperature matched ambient temperature, no voltage signal was generated (Fig. 6b). When a beaker with a small amount of hot water was placed, synchronous yet independent responses were triggered: a voltage change of ~ 200 μV and a resistance change of ~ 45% (Fig. 6c), confirming parallel temperature–pressure detection. Under identical pressure conditions, the pressure response times remained nearly constant across different temperatures, indicating minimal cross-sensitivity. Placing a large-volume hot-water beaker produced a similar voltage change (~ 200 μV) but a larger resistance shift (70%) due to the increased weight (Fig. 6d). Furthermore, under constant temperature, the temperature response times remained consistent despite varying pressures, demonstrating excellent decoupling between the two sensing modalities.

The same experiment was repeated with the sensor submerged underwater. The cold-water beaker again induced only a ~ 45% resistance change, without any voltage signal (Fig. 6e). The small-volume hot-water beaker triggered simultaneous voltage and resistance responses (Fig. 6f), verifying retained dual-mode operation in aquatic environments. The large-volume hot-water beaker produced a pronounced resistance increase while maintaining a stable voltage signal (Fig. 6g). Throughout these tests, pressure variations showed no significant effect on temperature sensitivity, and temperature changes did not alter pressure sensitivity. Comparing results from air (Fig. 6b–d) and underwater (Fig. 6f–h) environments reveal that the sensor maintains stable and consistent output signals in both conditions, demonstrating robust dual-mode amphibious sensing capability with effectively decoupled temperature and pressure responses.

**Fig. 7:**
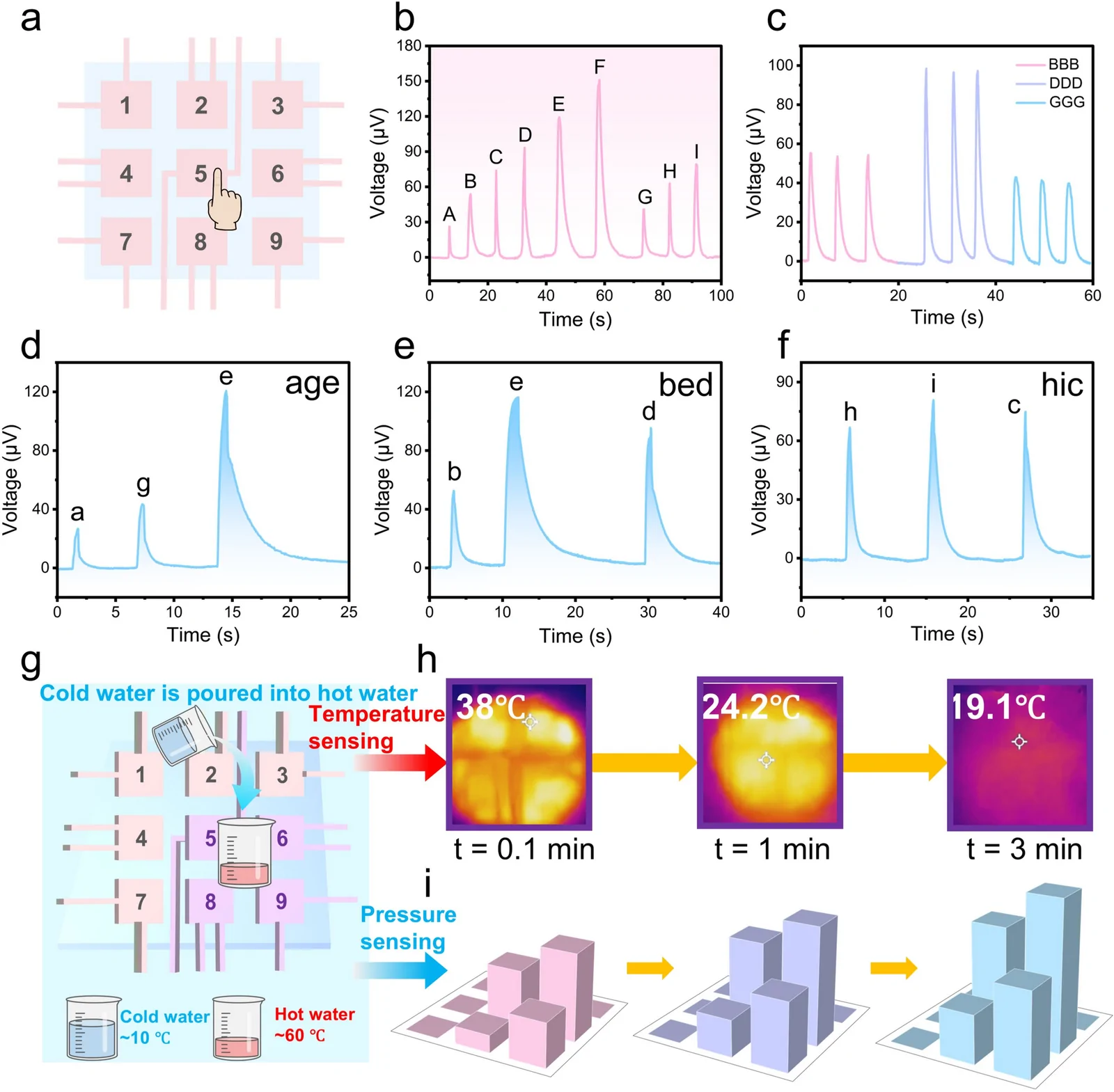
Configuration and dual‑mode sensing performance of an underwater 3 × 3 sensor array: **a** Schematic illustration of the array architecture, with each cell consisting of vertically stacked Bi_2_Se_3_/CP films; the number of stacked layers varies by position (2–12 layers). **b** Electrical signal patterns (denoted A–I) generated by finger touches at different positions (1–9) of the submerged array. **c** Signal stability and repeatability: voltage responses from three consecutive touches at the same position. **d–f** Demonstration of touch‑encoded word recognition: sequential touches at positions (1,7,5), (2,5,4), and (8,9,3) produce distinct signal sequences corresponding to the words “age”, “bed”, and “hic”, respectively. **g** Dynamic monitoring of simultaneous temperature and pressure variations using the sensor array. Spatiotemporal mapping of **h** temperature distribution and **i** pressure-induced resistance changes derived from the dynamic stimulation in **g**

As shown in Fig. S19, the output voltage of the sensor rises from 0.15 to 5.20 mV as the ∆*T* increases from 10 to 50 K. Notably, at a fixed ∆*T*, the voltage remains nearly constant under applied pressures ranging from 10 to 60 kPa, confirming that the voltage signal depends solely on ∆*T* and is independent of the applied pressure. These results demonstrated that the sensor can cleanly distinguish between thermal and pressure stimuli without cross-interference, establishing its fully decoupled dual-mode amphibious detection capability. Owing to its excellent dual-mode sensing and signal-decoupling performance, the sensor holds promising potential for wearable devices and electronic skin. To further illustrate this, a 3 × 3 sensor array was fabricated by stacking Bi_2_Se_3_/CP films with varying layer counts (Fig. 7a). The array was fixed to the bottom of a water-filled container to simulate underwater multimodal perception. As illustrated in Fig. 7b, each array position (1–9) produces a distinct output signal (labeled A–I). Finger touches on different positions of the submerged sensor array generate position-dependent thermoelectric voltage signals. Repeated touches at the same position yield highly consistent outputs (Fig. 7c), demonstrating excellent repeatability. By sequentially touching different designed positions, distinct signal sequences corresponding to specific words can be produced. For example, touching positions 1–7-5, 2–5-4, and 8–9-3 encode the words “age”, “bed”, and “hic”, respectively (Fig. 7d–f).

The underwater sensor array further demonstrates its capacity for real-time, dual-mode monitoring of spatially varying thermal and mechanical stimuli. As shown in Fig. 7g, a beaker partially filled with hot water (~ 60 °C) was placed over positions (5,6,8,9) of the submerged array. Cold water (~ 10 °C) was then slowly added to the beaker, gradually lowering its temperature to ~ 20 ºC. Throughout this process, the concurrent decrease in thermal load and increase in hydrostatic pressure (due to the added water mass) were monitored synchronously by the array via corresponding voltage and resistance changes at the affected positions (e.g., Fig. 7g–i). This result demonstrates that the sensor array independently and simultaneously tracks dynamic temperature and pressure variations in an aquatic setting, highlighting its significant potential for advanced wearable sensing and e-skin applications in underwater environments. The waterproof and sensing performances demonstrated in this work were all obtained under ambient-pressure shallow-water conditions, while the stability of the interfacial wetting state and the long-term operational reliability under deep-sea high-pressure environments require further investigation.

### Electromagnetic Shielding Performance in Air and Underwater

**Fig. 8:**
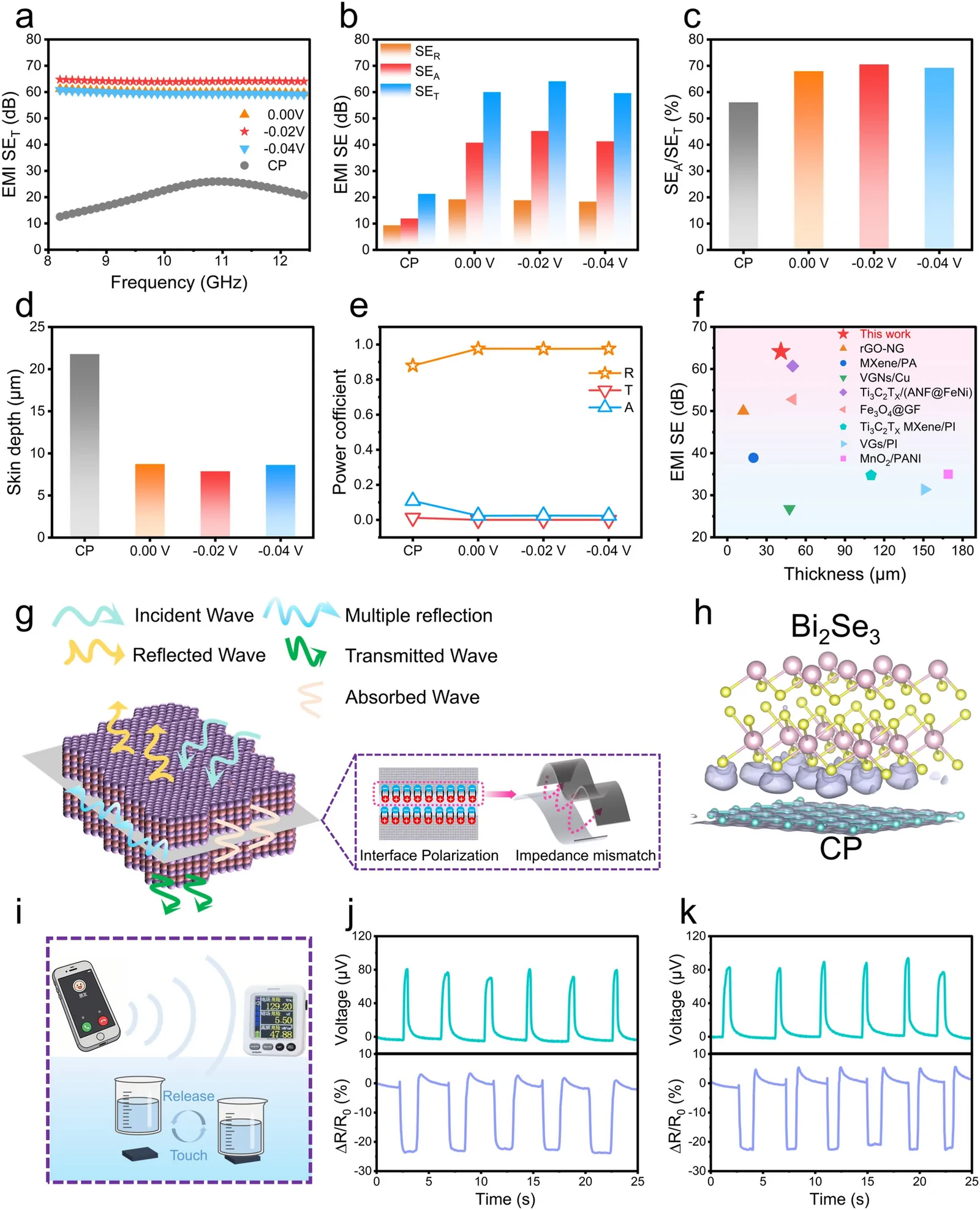
EMI shielding performance of the Bi_2_Se_3_/CP film, and its underwater sensing stability under ambient EMI: **a** SE_T_ of Bi_2_Se_3_/CP deposited at different potentials (0.00, − 0.02, and − 0.04 V) compared to pristine CP over the X-band (8.2–12.4 GHz). **b** Average SE_T_, SE_A_, and SE_R_ in the X-band. **c** SE_A_/SE_T_ ratio for CP and Bi_2_Se_3_/CP films. **d** *δ* of films deposited at various potentials. **e** R, A, and T coefficients for the CP and Bi_2_Se_3_/CP films. **f** Comparison of SE_T_ and thickness between this work and previously reported shielding materials. **g** Schematic diagram of EMI shielding mechanism within the Bi_2_Se_3_/CP composite. **h** Charge density difference of the Bi_2_Se_3_/CP interface calculated by DFT (Pink: Bi, yellow: Se, green: C, purple: charge accumulation, gary: depletion). **i** Experimental setup for evaluating the output-signal stability under simulated EMI from a mobile phone in calling and standby modes in an underwater environment. Output voltage and resistance signals when the sensor is approached by a mobile phone in **j** standby mode (electric field: 1.32 V/m; magnetic field: 0.00 μT; power density: 0.00 μW cm^−2^) and **k** calling mode (electric field: 129.20 V m^−1^; magnetic field: 5.50 μT; power density: 47.88 μW cm^−2^.)

In complex electromagnetic environments, electromagnetic interference (EMI) degrades the sensor performance by inducing charge accumulation and magnetic field coupling, which distorts temperature and pressure signals [56, 57]. This is particularly critical for conductive materials, where high sensitivity coexists with susceptibility to external magnetic fields, leading to signal drift [58, 59]. Therefore, intrinsic EMI shielding capability is essential for reliable operation. Beyond sensing, the Bi_2_Se_3_/CP films exhibit excellent EMI shielding performance. As shown in Fig. 8a, the composite films significantly outperform pristine CP. At the optimal deposition potential of − 0.02 V, the total shielding effectiveness (SE_T_) exceeds 62 dB across the entire X-band, a 3.2-fold improvement over CP (~ 20 dB). SE_T_ is the combined contribution of three components: reflection loss (SE_R_), absorption loss (SE_A_), and internal multiple reflection loss (SE_M_) [60, 61]. When SE_T_ exceeds 15 dB, SE_M_ can be neglected. The SE_R_ value of the deposited samples exceeded 10 dB, confirming impedance mismatch between the material and free space [62]. The shielding mechanism is absorption-dominant: the optimal film shows average SE_A_, SE_R_, and SE_T_ values of 45.2, 18.9, and 64.1 dB, respectively, attenuating > 99.9% of incident energy (Fig. 8b). The SE_A_/SE_T_ ratio reaches 70.5%, indicating a 1.3‑fold enhancement in absorption contribution compared to CP (56.1%) (Fig. 8c).

Skin depth (*δ*) is a critical parameter for assessing EMI shielding performance, defined as the depth at which the wave intensity attenuates to 1/*e* (~ 37%) of its initial value. The quantitative equation for *δ* is as follows [63]:

5 $$\delta = 8.686 \times \frac{d}{{SE}_{A}}$$

where *d* is the thickness. The film deposited at − 0.02 V exhibits the smallest *δ* of 7.87 μm (Fig. 8d), attributable to the optimized synergy between thickness and conductivity. The average reflection (R), absorption (A), and transmission (T) coefficients for the CP and Bi_2_Se_3_/CP films are shown in Fig. 8e). The near‑zero T for all samples confirms that incident waves are almost entirely attenuated within the material. Although the high R over 0.9 indicates that over 90% of incident energy is initially reflected at the interface, the dominant shielding mechanism remains absorption-controlled. This is because the reflected portion does not penetrate the material, while the residual waves that enter undergo strong absorption via polarization and multiple-scattering losses inside the composite. Thus, despite the high reflectivity, effective attenuation is governed by absorption processes after initial reflection. Compared with other reported materials, the Bi_2_Se_3_/CP composite demonstrates competitive shielding performance relative to its thickness (Fig. 8f) [64–71].

The shielding mechanism arises from its layered structure and interfacial design. Bi_2_Se_3_ exhibits an intrinsic layered crystal structure (Fig. S20), which promotes multiple scattering and polarization loss of electromagnetic waves. As illustrated in Fig. 8g, when incident waves interact with the Bi_2_Se_3_/CP film, the high conductivity of Bi_2_Se_3_ and its pronounced impedance mismatch with the surrounding medium cause immediate reflection of a portion of the radiation. The remaining waves penetrate the material, where the extensive interfacial network induces repeated scattering and gradual energy dissipation. During propagation, free charge carriers are captured by structural defects, leading to localized space-charge accumulation. DFT calculated charge-density difference maps (Fig. 8h) reveal non-uniform carrier distribution of carriers at the Bi_2_Se_3_/CP interface and defect sites, generating dipole moments and local fields thereby inducing dipole polarization, where the purple and gray regions represent charge accumulation and depletion areas, respectively. This combined scattering and polarization mechanism underpins its effective electromagnetic shielding performance [72].

To evaluate its sensing stability under real‑world EMI, the signal output of the sensor was monitored in an underwater environment while simulating field variations from mobile phone in call and standby mode (Fig. 8i). Owing to the relatively high electrical conductivity and dielectric loss of seawater, high-frequency electromagnetic waves exhibit limited skin depth and undergo significant attenuation during propagation in seawater [73, 74]. Therefore, far-field electromagnetic interference is generally unlikely to be a major source of disturbance for underwater sensors operating in marine environments. Consequently, the EMI shielding performance of this device should not be primarily interpreted as an advantage against far-field underwater interference. More specifically, the practical significance of this property is mainly reflected in two aspects. First, in air, the excellent EMI shielding capability helps reduce the disturbance of external electromagnetic noise during signal acquisition and transmission, thereby improving the stability and reliability of the output signals. Second, in practical underwater electronic systems, although seawater naturally attenuates high-frequency far-field electromagnetic waves, near-field electromagnetic interference generated by surrounding electrical components may still exist and affect the sensor output. Experiments confirm that under common EMI sources, including standby-mode radiation (900 MHz/1.8 GHz) and communication-band signals (2.4 GHz), the sensor maintains stable voltage and resistance outputs in both air and underwater environments (Figs. 8j, k and S21a–d). This stability arises from the combined effect of its high intrinsic shielding effectiveness and the low energy levels of ambient electromagnetic waves, which are insufficient to induce notable thermal fluctuations or excite carriers across the Bi_2_Se_3_ bandgap. Consequently, the excellent electromagnetic interference shielding performance reflects superior electromagnetic compatibility, enabling stable signal output across different environments and providing a reliable platform for dual-mode monitoring.

### Hydrophobicity, Flexibility, and Stability

**Fig. 9:**
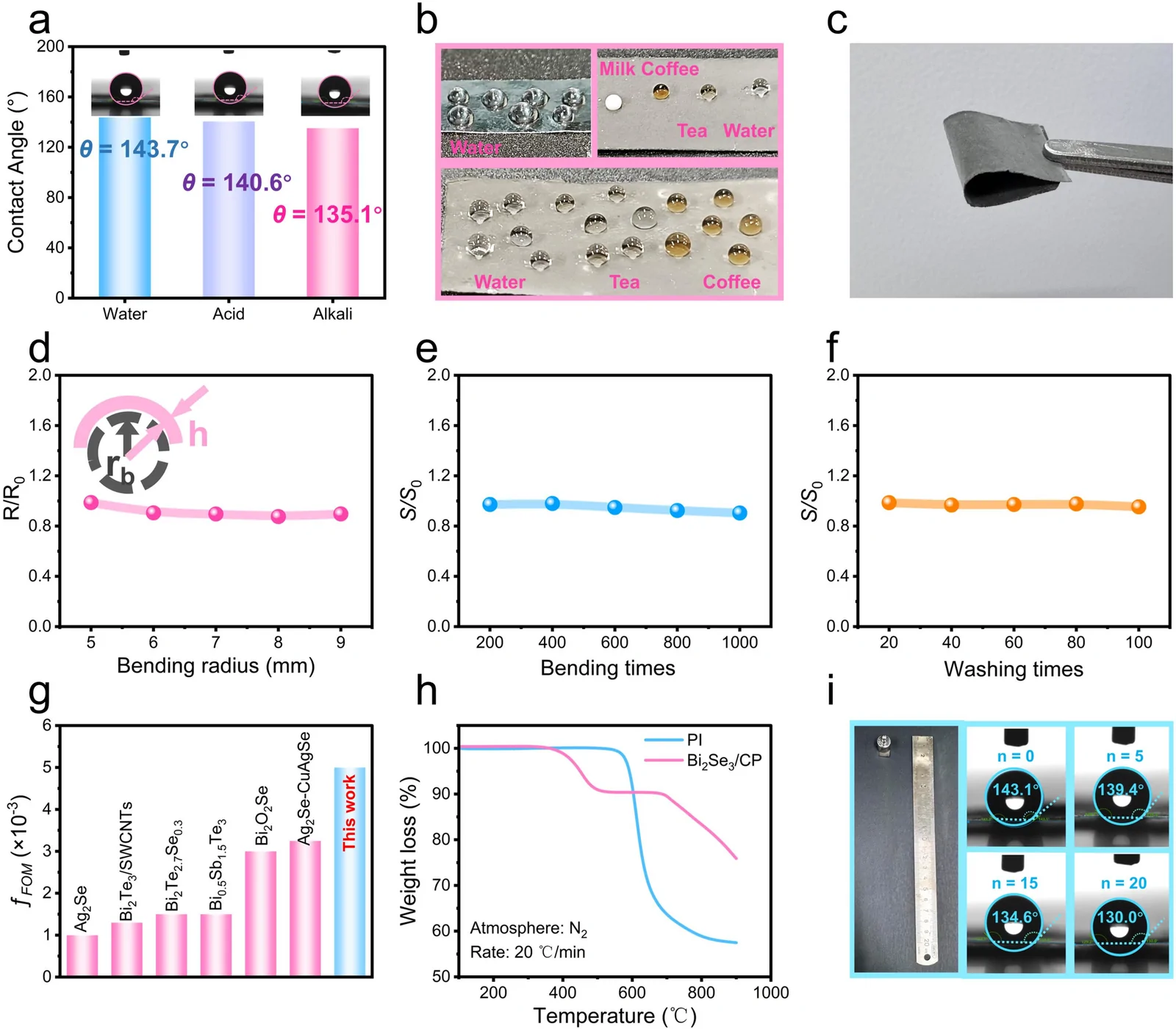
Mechanical flexibility, environmental durability, and thermal stability of the Bi_2_Se_3_/CP film: **a** Contact angles of the film measured with aqueous solutions at pH 4, 7 and 10. **b** Comparison of contact angles of common liquids (water, coffee, tea, milk) on CP and Bi_2_Se_3_/CP films. **c** Photograph of the film under manual bending. **d** Normalized resistance change (*R/R*_0_) as a function of bending radius ( *r*_b_), where *R*_0_ and *R* denote the resistance in the flat and bent state, respectively. **e** Normalized Seebeck coefficient (*S/S*_0_) after 200 to 1000 bending cycles, where *S*_0_ and *S* represent the Seebeck coefficients in the flat and bent states, respectively. **f** Normalized Seebeck coefficient after 20–100 immersion cycles in deionized water. **g** Comparison of the ƒ_FOM_ for the Bi_2_Se_3_/CP film and other Bi_2_Se_3_-based films reported. **h** Thermogravimetric (TG) curves of the Bi_2_Se_3_/CP film and PI measured under inert atmosphere. **i** Scratch resistance test: optical image of the film surface after abrasion with 800-grit silicon carbide paper and corresponding water contact angles measured after increasing abrasion cycles

To demonstrate the stability of the composite film in underwater applications, contact angle tests conducted using solutions of different pH values (pH = 4, 7, 10) yielded angles of 143.7°, 140.6°, and 135.1°, respectively (Fig. 9a), confirming maintained hydrophobicity and chemical stability across acidic to alkaline conditions. The film also exhibited superior repellency against common liquids (water, coffee, tea, milk), with consistently higher contact angles than pristine CP (Fig. 9b). The mechanical and environmental durability of the Bi_2_Se_3_/CP films was systematically evaluated through bending and immersion tests to simulate practical wearable conditions. As shown in Fig. 9c, the film exhibits good flexibility under manual bending. Quantitative curvature-dependent resistance measurements (*R/R*_0_, where *R* and *R*_0_ are the resistances under bending and in the flat state) were taken by conforming the film to glass tubes with defined radius (Fig. 9d). The resistance change remained below 10% even at a bending radius of 5 mm, indicating excellent flexibility. Additionally, cyclic bending tests (200–1000 cycles at 90°) revealed minimal variation in the *S*, with fluctuations within 10% of its initial value, confirming robust stability against repeated deformation (Fig. 9e).

Furthermore, the film also exhibited excellent water immersion stability. After 100 immersion cycles in deionized water, its resistivity retained > 95% of the original value, with no observable detachment or degradation (Fig. 9f). For a meaningful comparison of flexibility across materials, thickness must be considered. Peng et al. [75] proposed a flexibility figure of merit ƒ_FOM_ = *h*/2*r*, where *r* is the critical radius and *h* is the film thickness. As shown in Fig. 9g, the Bi_2_Se_3_/CP film exhibits the highest ƒ_FOM_ value among recently reported Bi_2_Se_3_-based and selenide-based materials [76–79]. Figure 9h shows the thermogravimetric curve of the composite film during heating. No mass loss was observed up to 400 ºC, indicating high thermal stability within this range. Between 400 and 500 °C, a sharp weight reduction of ~ 12% is observed, attributable to the desorption of residual adsorbed species (e.g., surface water) and the onset of partial decomposition of the Bi_2_Se_3_ phase. The weight then stabilizes between 500 and 700 °C range, likely due to the stabilizing effect of the CP, which may inhibit further Se volatilization through interfacial interactions and its own inherent stability. Above 700 °C, the CP undergoes pyrolysis and the residual Bi_2_Se_3_ decomposes completely, resulting in further mass loss. The flame‑retardant behavior of the Bi_2_Se_3_/CP composite film was evaluated using an alcohol‑lamp flame test, with PI as a reference due to its known flame‑retardant properties. Figure S22a illustrates the experimental setup. Infrared thermal images recorded as the films approached the outer flame reveal distinct thermal responses: the temperature of the Bi_2_Se_3_/CP film rose from 37.4 to 77.9 °C as the distance decreased from 3 cm to contact, whereas PI exhibited a much steeper rise from 33.9 to 131.1 °C under the same conditions (Fig. S22b, c).

Furthermore, direct flame exposure tests were conducted (Fig. S23). The Bi_2_Se_3_/CP film showed no ignition or significant deformation after 200 s in the flame, remaining largely intact (Fig. S23a). In contrast, the PI film bent and carbonized within 1.5 s, ignited at 3.5 s, and sustained burning (Fig. S23b). Additional tests in an oxygen‑index apparatus (Fig. S24) confirmed that the film did not ignite below 50% O_2_ concentration even after 30 s of exposure. At 50% O_2_, only slight surface decomposition occurred, which ceased immediately upon flame removal. These results collectively demonstrate that the Bi_2_Se_3_/CP composite film possesses notable thermal stability and flame‑retardant characteristics, which are advantageous for applications requiring operation in high‑temperature or flammable environments.

Mechanical durability was evaluated through abrasion and ultrasonic tests. Scratch testing was using 800-grit silicon carbide sandpaper under a 10-g load for 15 friction cycles (*n* = 15) left visible residue on the abrasive, and the water contact angle showed only minor variation (Fig. 9i), indicating a dense, adherent coating. Furthermore, samples subjected to 3 h of ultrasonication (53 kHz) showed no noticeable detachment (Fig. S25a, b), and their conductivity and Seebeck coefficient (Fig. S25c, d) remained largely unchanged, confirming robust interfacial integrity. The film also displays controllable Joule-heating capability. Under applied voltages of 0.1, 1, and 2 V, steady-state temperatures of 38.6, 40.4, and 43.7 ºC were achieved (Fig. S26a), respectively. Repeatable heating and cooling cycles between 0.1 and 2 V (Fig. S26b) and consistent performance over six consecutive cycles at 0.1 V (Fig. S26c) demonstrate reliable thermal cyclability. This feature, coupled with low‑voltage operation, supports its integration as a safe, wearable heater.

## Conclusions

In response to the application demands in high-humidity environments such as marine resource exploration and underwater environmental monitoring, flexible sensors face considerable challenges in achieving stable operation across different media (air and water) and independently decoupling signals from multiple physical quantities such as temperature and pressure. This study constructed a Bi_2_Se_3_ layer with a pyramid structure on the surface of flexible CP via pulsed electrochemical deposition. By optimizing the deposition potential to − 0.02 V, the film achieved an electrical conductivity of 779.3 S cm^−1^, a Seebeck coefficient to − 36.9 μV K^−1^, and a power factor of 106.0 μW m^−1^ K^−2^. The unique pyramidal interface confers inherent hydrophobicity (contact angle 143.7°) and a piezoresistive effect, enabling decoupled detection of temperature and pressure signals in both aerial and underwater environments. The material also exhibits remarkable electromagnetic shielding performance, flexibility, and flame retardancy, and excellent stability, meeting the requirements for all-weather operation in aquatic and submerged conditions. This developed sensor has been successfully applied in respiratory monitoring, underwater micro-disturbance detection, and human motion tracking, providing an innovative solution for wearable dual-mode amphibious sensing.

## Supplementary Information

Below is the link to the electronic supplementary material.

Supplementary file1 (DOCX 8227 kb)
