# Supplementary material for: Stack of Bi2Se3/Carbon Films with Pyramid Interface for Dual-Mode Temperature–Pressure Sensing in Aquatic Environments
Source: Nanomicro Lett. 2026 Jun 22;18:409. doi: 10.1007/s40820-026-02254-0 (PMC13287202; doi:10.1007/s40820-026-02254-0)
Supplement: Supplementary file 1 — Supplementary file1 (DOCX 8227 kb) [file 40820_2026_2254_MOESM1_ESM.docx]

Supporting Information for

**Stack of Bi_2_Se_3_/Carbon Films with Pyramid Interface for Dual-Mode Temperature-Pressure Sensing in Aquatic Environments**

Yan Xu^1^, Xuefei Zhang^2^, Size Lou^1^, Zhe Tang^1^, Dongmei Xie^1^, Chuanrui Zhang^1^, Mengran Chen^1^, Heng Liu^1,3,*^, Chuan Sun^4^, Yixiang Ou^5^, Peng-an Zong^1,*^

^1^ College of Materials Science and Engineering, Jiangsu Collaborative Innovation Center for Advanced Inorganic Function Composites, Nanjing Tech University, Nanjing 211816, P. R. China

^2^ State Key Laboratory of New Ceramics and Fine Processing, School of Materials Science and Engineering, Tsinghua University, Beijing 100084, P. R. China

^3^ Advanced Institute for Materials Research (WPI-AIMR), Tohoku University, Sendai 980-8577, Japan

^4^ National Innovation Institute of Defense Technology, Academy of Military Sciences of the People’s Liberation Army of China, Beijing 100850, P. R. China

^5^ Joint Laboratory of Advanced Energy Materials and Intelligent Equipment, Beijing Academy of Science and Technology, Beijing 100000, P. R. China

*Corresponding authors. E-mail: [pazong@njtech.edu.cn](mailto:pazong@njtech.edu.cn) (Peng-an Zong); [heng.liu.e1@tohoku.ac.jp](mailto:heng.liu.e1@tohoku.ac.jp) (Heng Liu)

**Supplementary Figures and Tables**


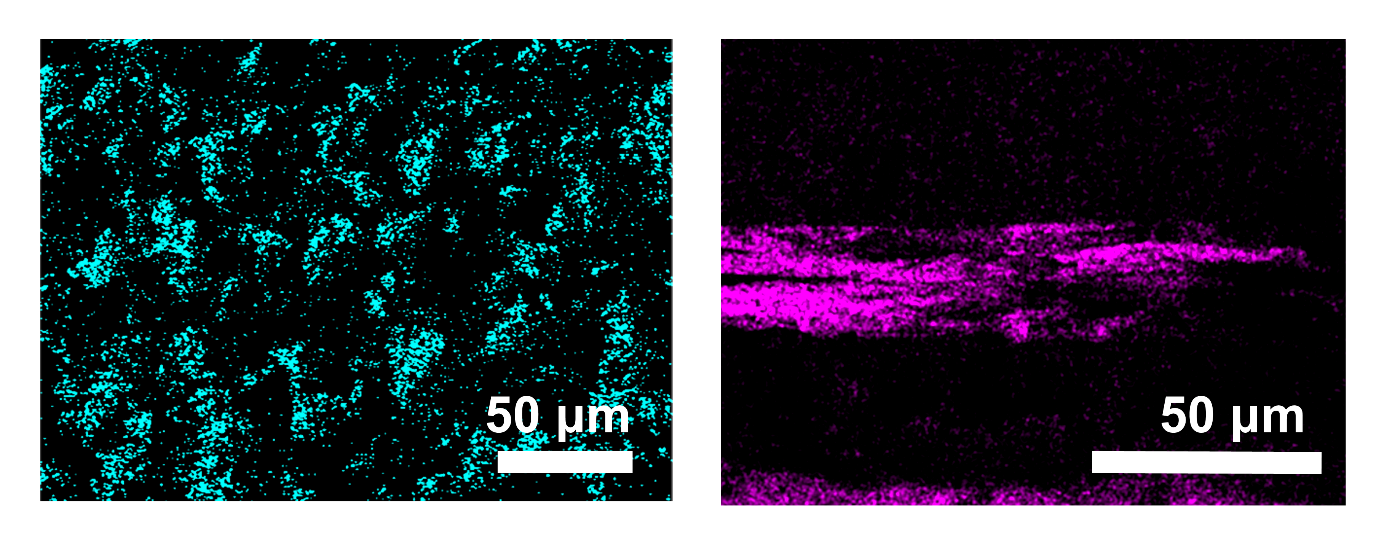


**Fig. S1** EDS spectrum of element carbon (C) on the **(a)** surface and **(b)** cross-section of the Bi_2_Se_3_/CP composite film.


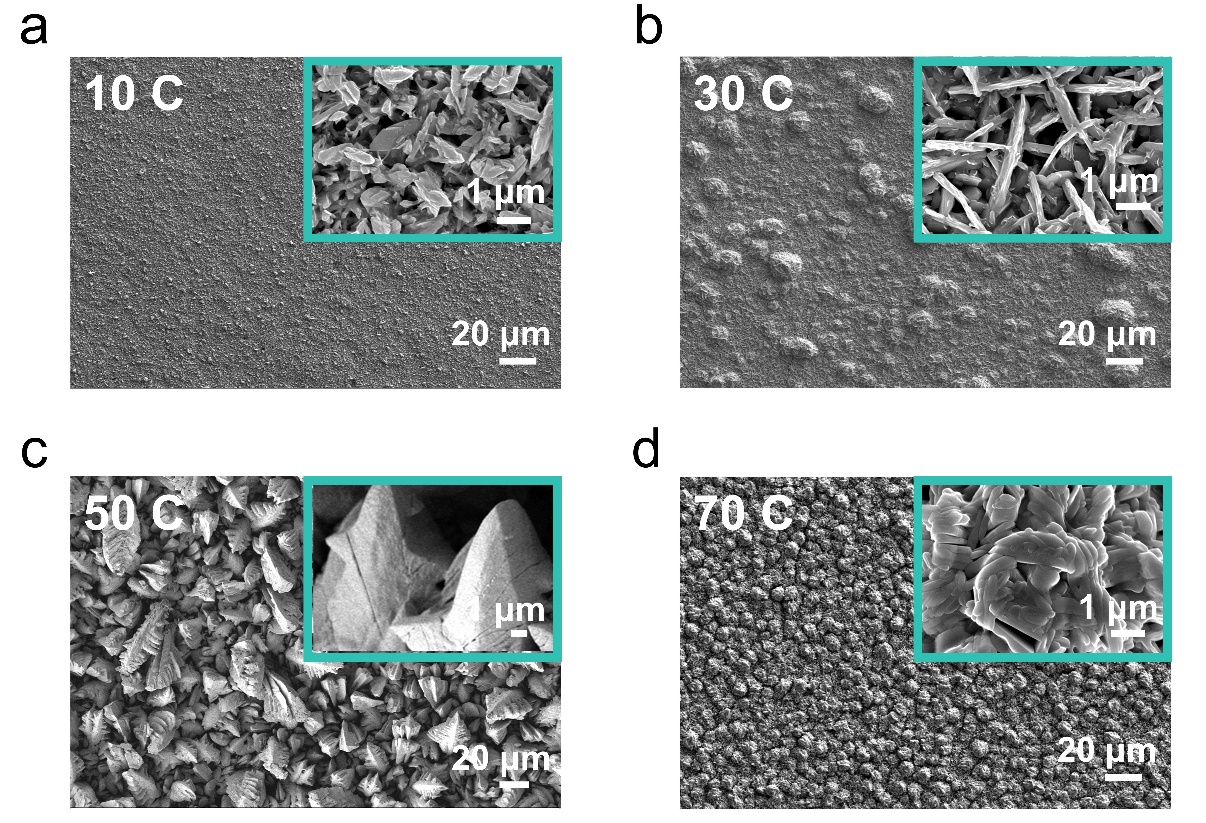


**Fig. S2** SEM images of the surface morphologies of Bi_2_Se_3_/CP films deposited with different coulombic quantities, together with their corresponding enlarged views. **a** 10 C. **b** 30 C. **c** 50 C. **d** 70 C.


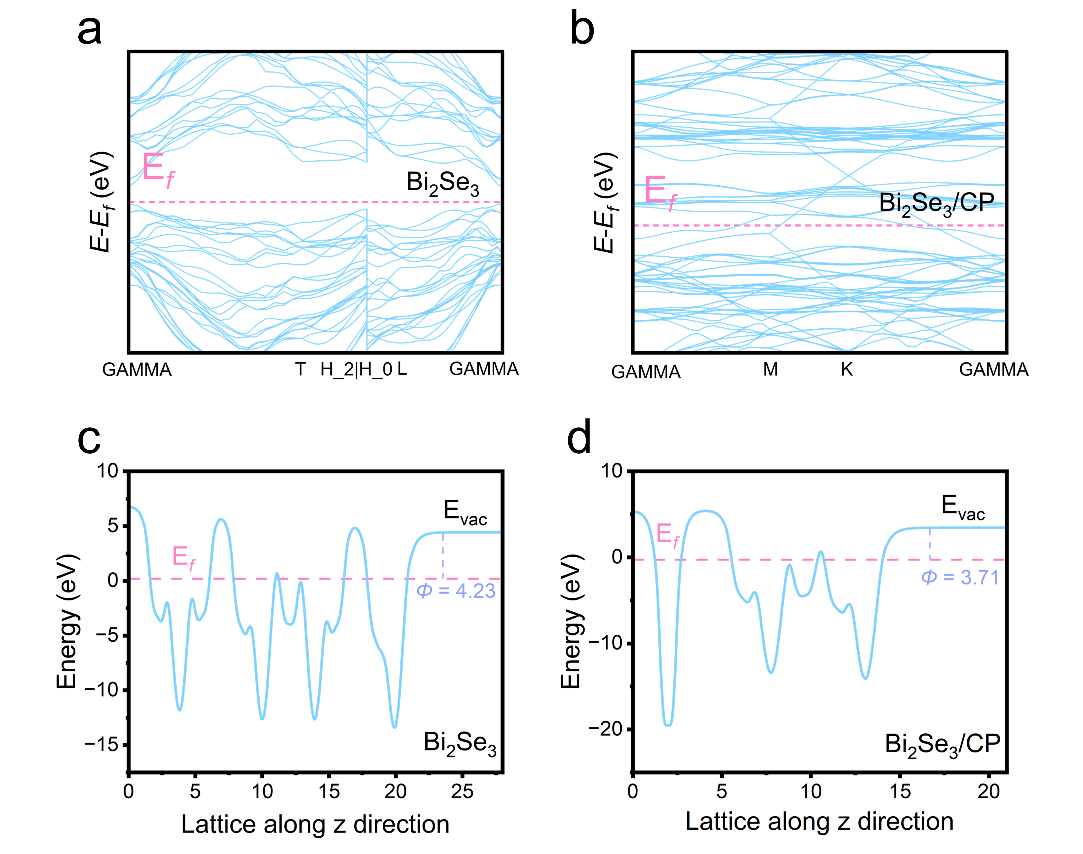


**Fig. S3 Electronic band structure and work function of Bi_2_Se_3_ and of Bi_2_Se_3_/CP. a, b** Calculated band structures of (a) pristine Bi_2_Se_3_ and (b) Bi_2_Se_3_/CP. **c, d** Corresponding Fermi level, vacuum level and work function(*Φ*) of (c) Bi_2_Se_3_ and (d) of Bi_2_Se_3_/CP.


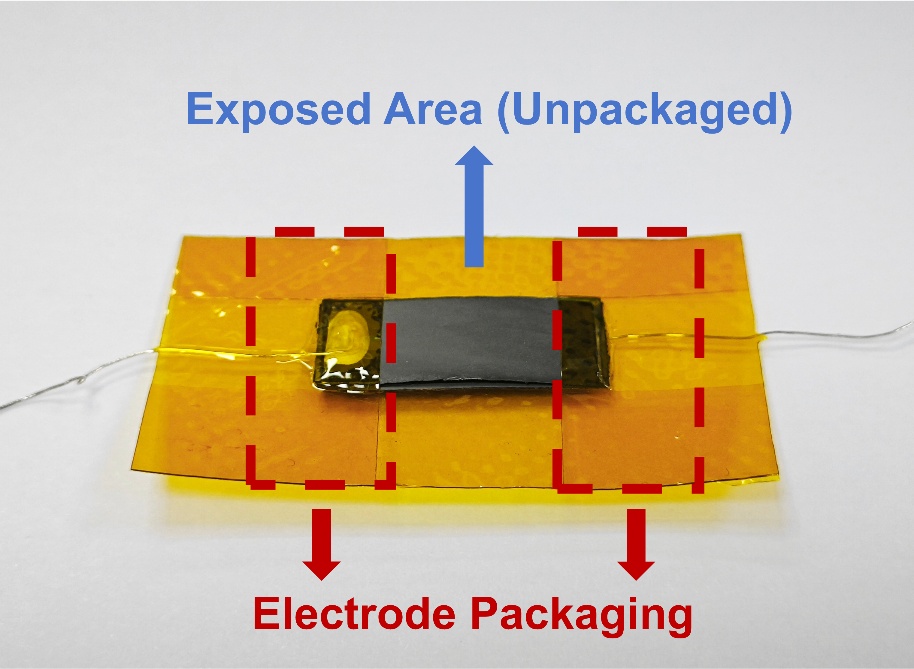


**Fig. S4 Configuration of the Bi_2_Se_3_/CP stacked film sensor for sensing tests.**

The sensor device consists of ten stacked Bi_2_Se_3_/CP films, each measuring 1 cm × 2 cm with a thickness of 50 ± 5 μm. Silver wires were attached to both ends of the stack using silver paste to establish electrical contacts, and the connection regions were encapsulated with polyimide (PI) tape to ensure mechanical stability and reliable electrical interfacing. One side of the device was further secured with PI to preserve structural integrity during testing. This configuration enabled stable and reproducible sensor operation throughout all measurements.


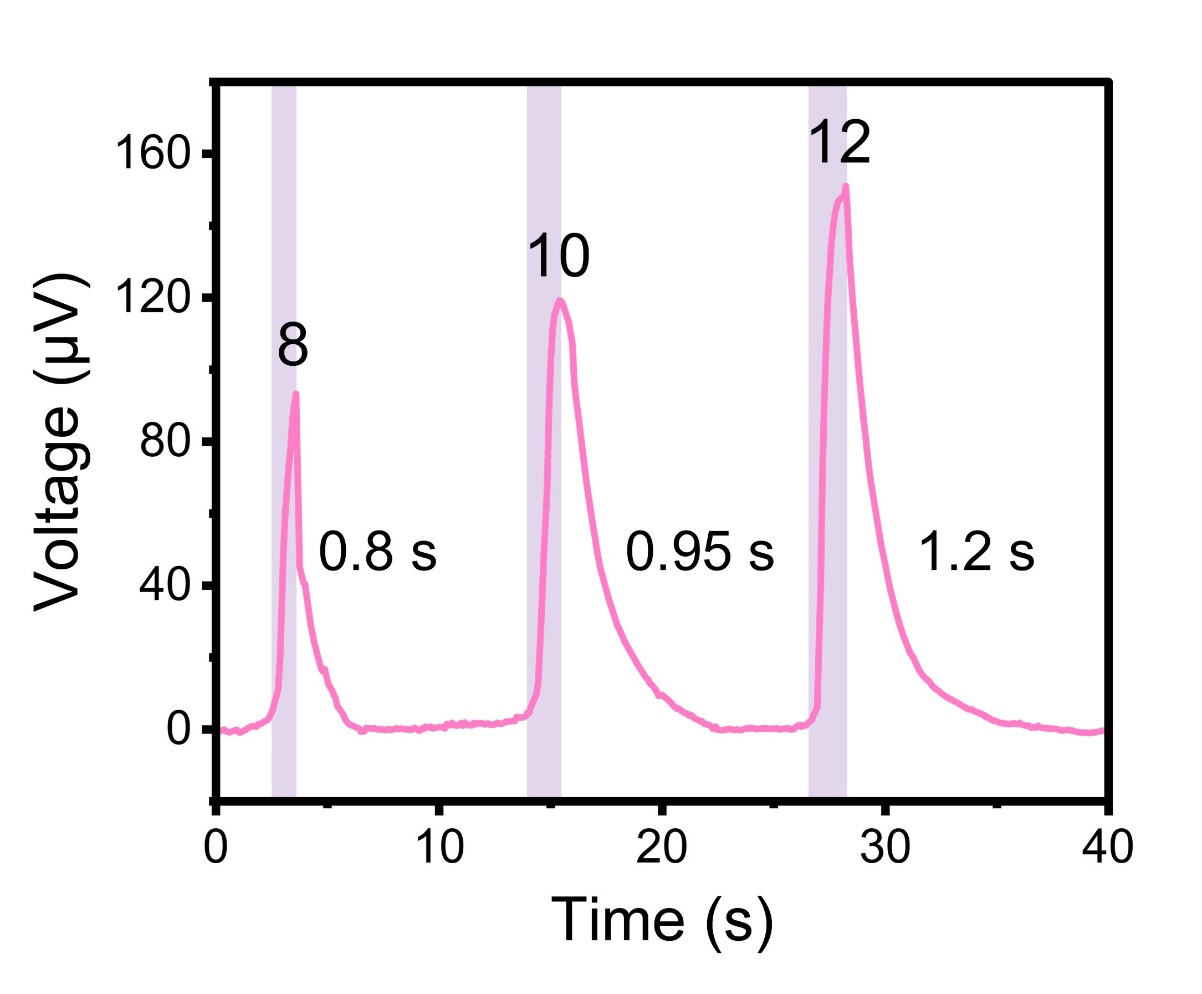


**Fig. S5 Comparison of the voltage outputs and response times of stacked devices with different layer numbers (8, 10, and 12) under the same temperature stimulus.**


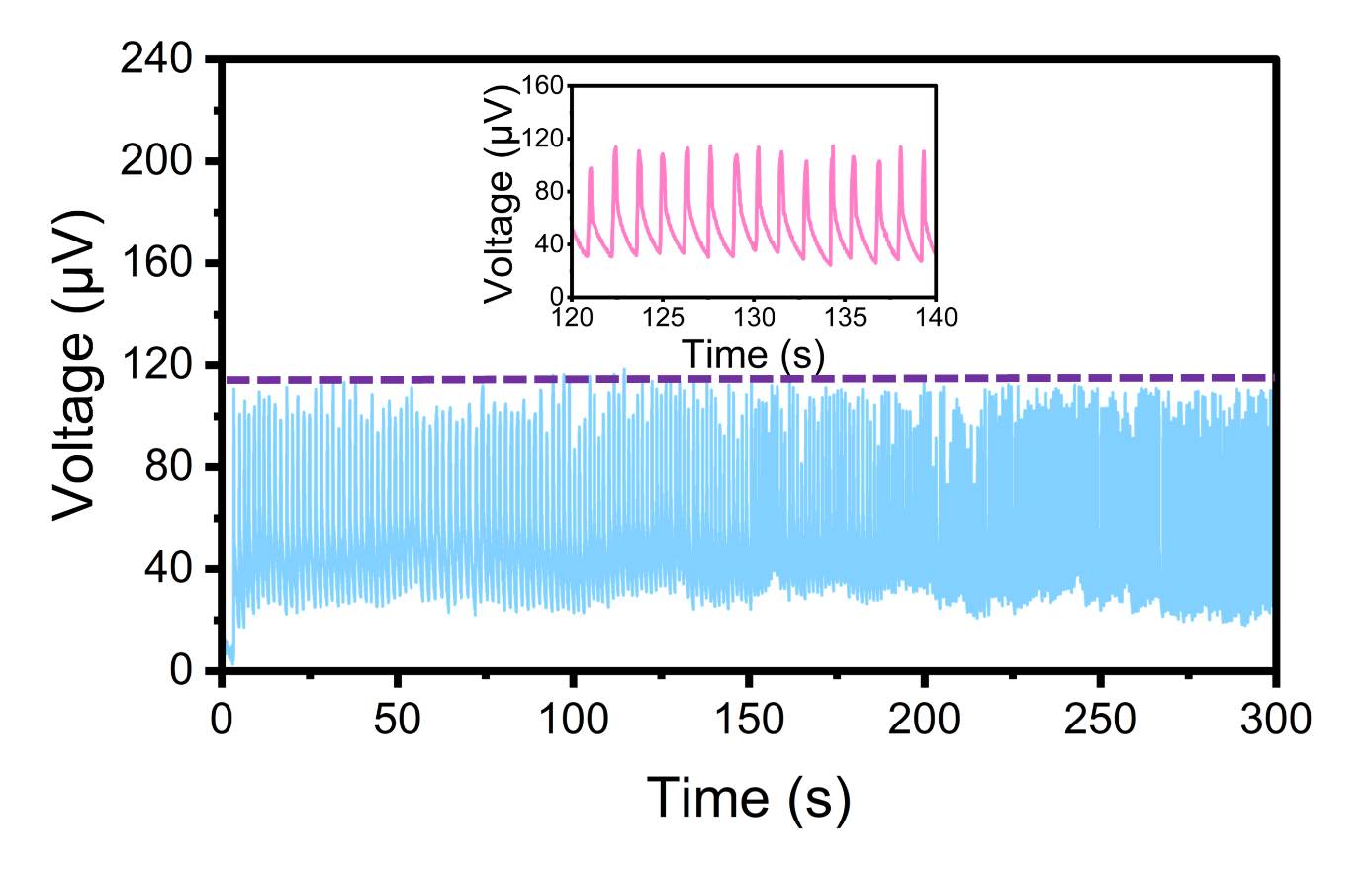


**Fig. S6 Voltage response of the sensor under continuous finger contact in air over 300 s.**

The inset provides an enlarged view of the response during 120-140 s, demonstrating excellent signal stability.


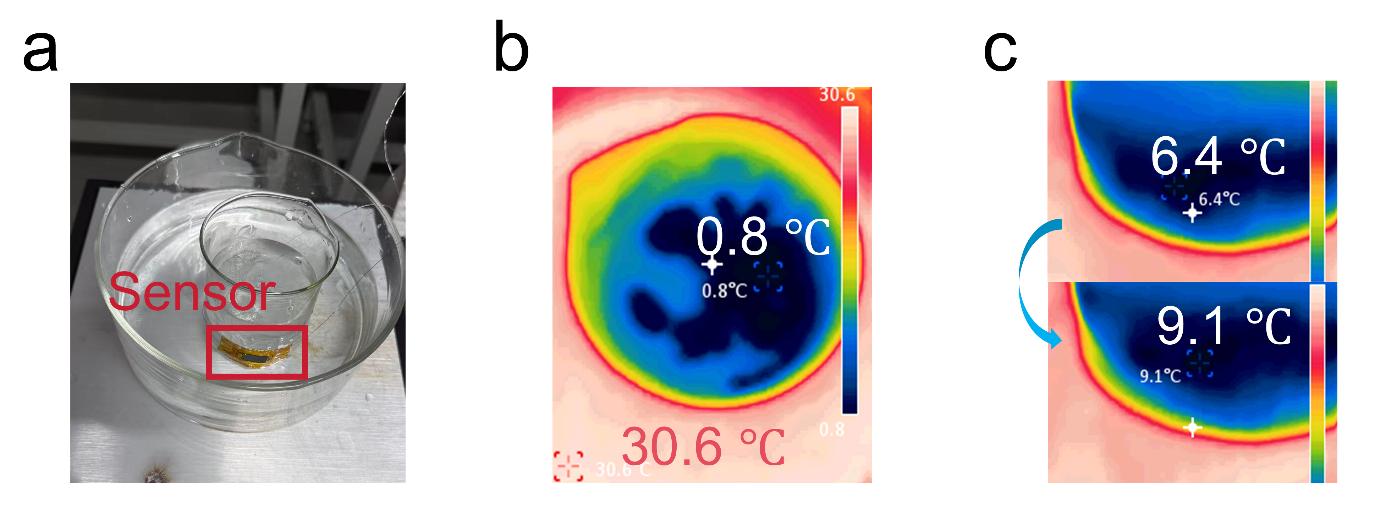


**Fig. S7 Experimental setup for evaluating the voltage response of the submerged sensor under controlled temperature differences.**

**a** Photograph showing the sensor attached to the outer wall of a beaker filled with an ice–water mixture. The beaker is subsequently immersed in hot water at various temperatures to establish a temperature gradient across the sensor. **b** Infrared thermal image captured immediately after immersion, showing the beaker wall at 0.8 °C, and the surrounding hot water at 30.6 °C. **c** Infrared thermal images recorded 15 s and 30 s after immersion, with the beaker wall temperature rising to 6.4 °C and 9.1 °C, respectively.


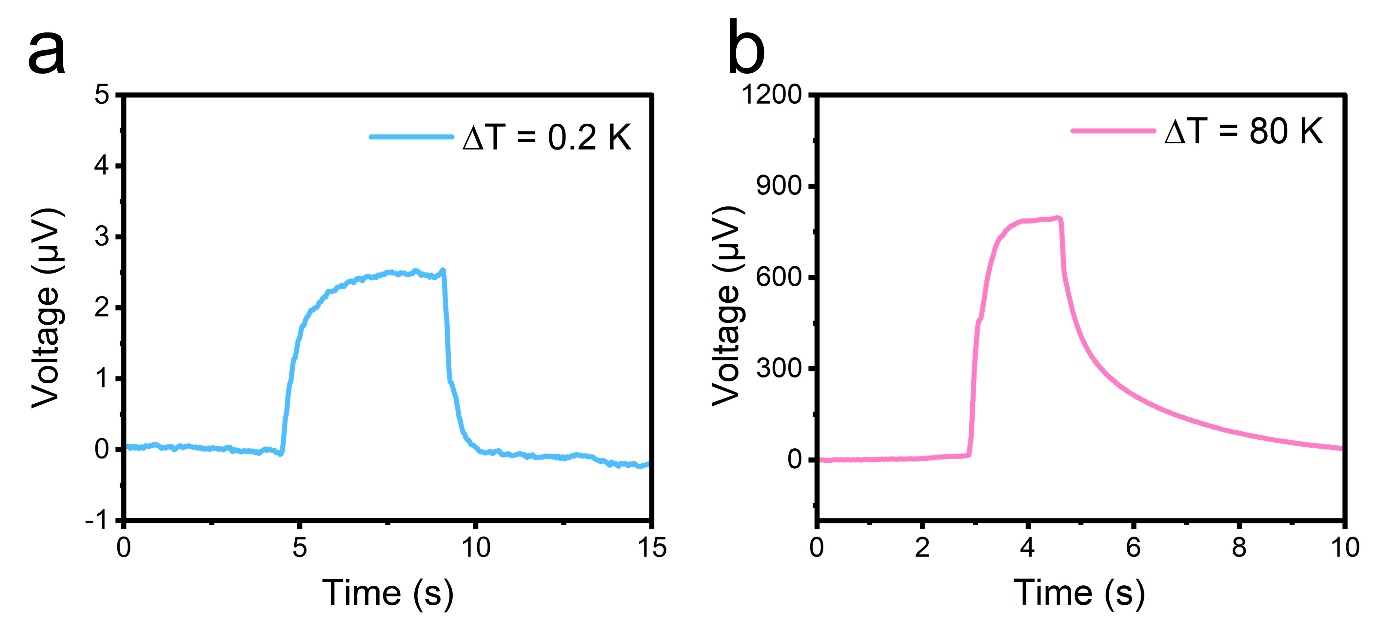


**Fig. S8 Detection limit tests of the device for temperature sensing.**

**a** Δ*T* = 0.2 K. **b** Δ*T* = 80 K.


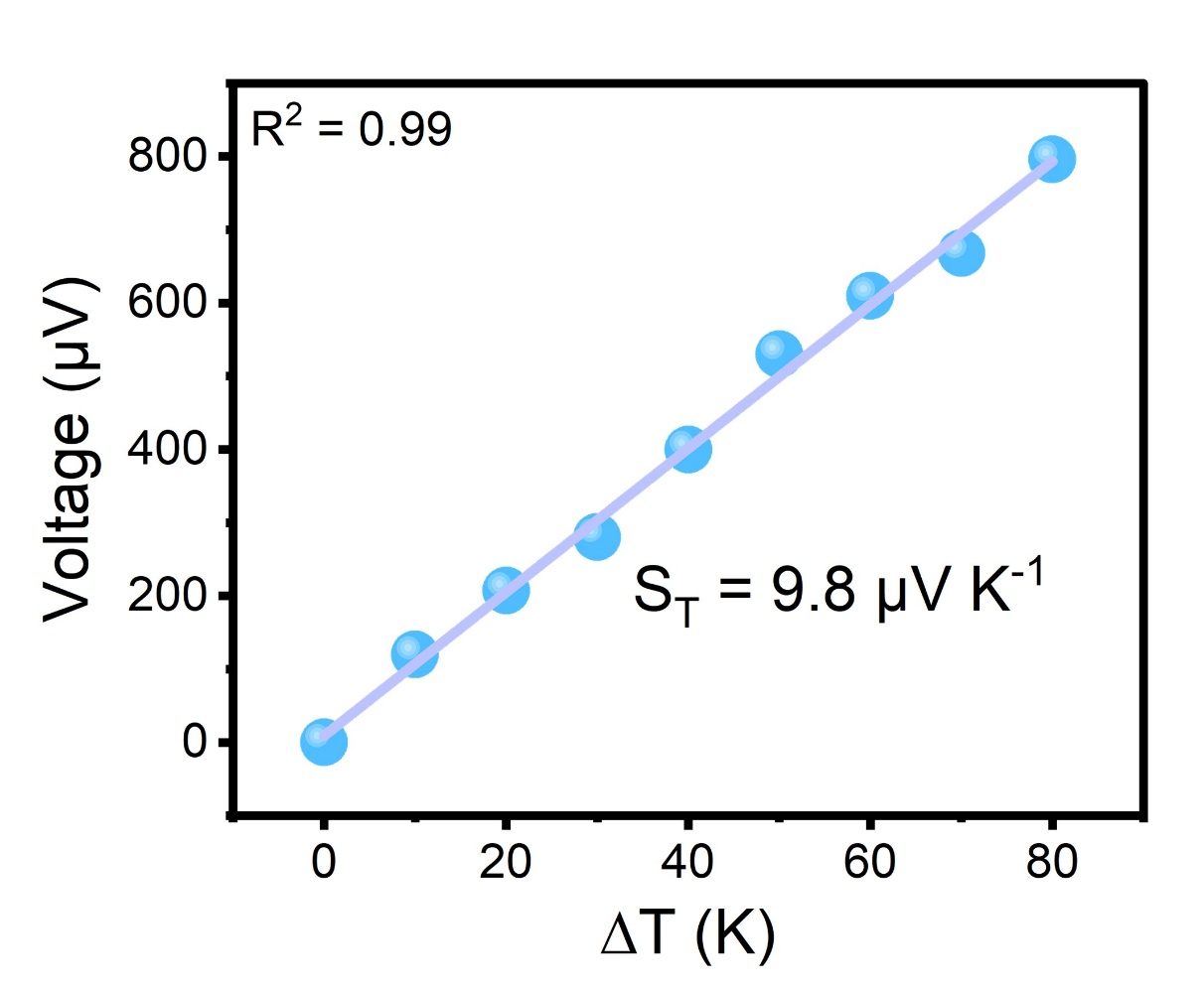


**Fig. S9 Voltage output *vs*. temperature difference curve.**


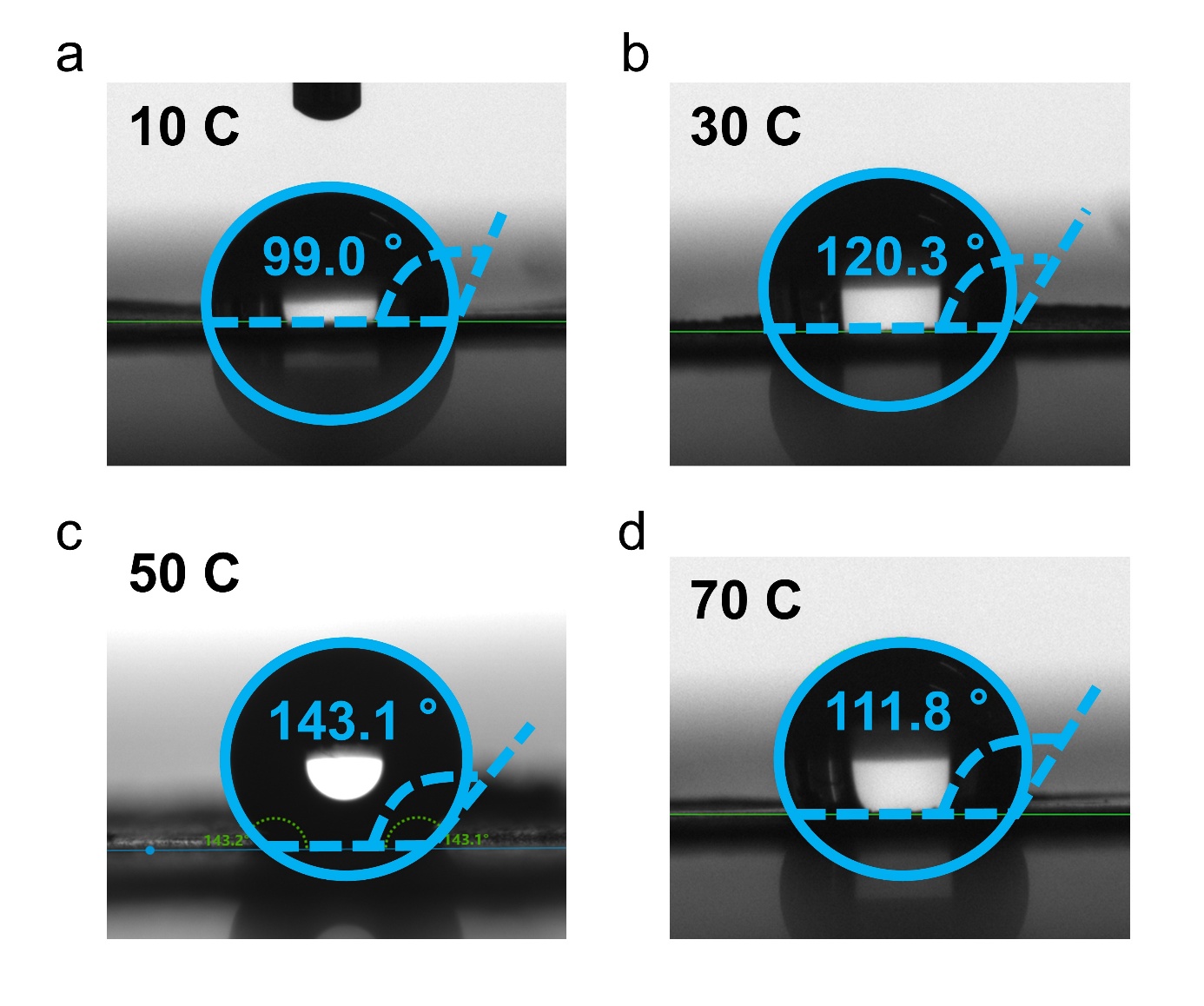


**Fig. S10** **Contact angles of Bi_2_Se_3_/CP films deposited with different coulombic quantities. a** 10 C. **b** 30 C. **c** 50 C. **d** 70 C

.
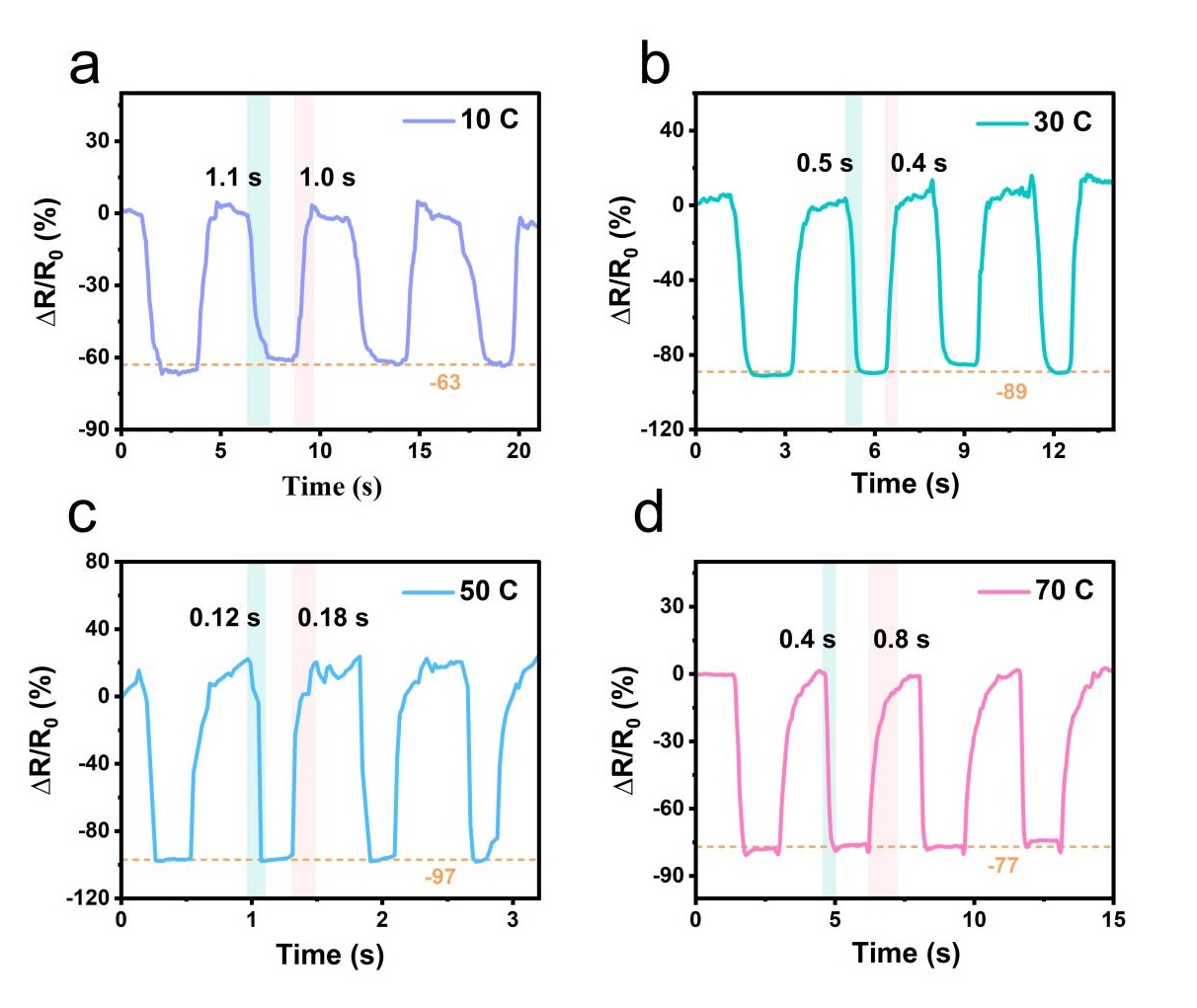


**Fig. S11** **Bi_2_Se_3_/CP films deposited with different coulombic quantities were stacked to assemble devices, which were then subjected to pressure sensing tests. a** 10 C. **b** 30 C. **c** 50 C. **d** 70 C.


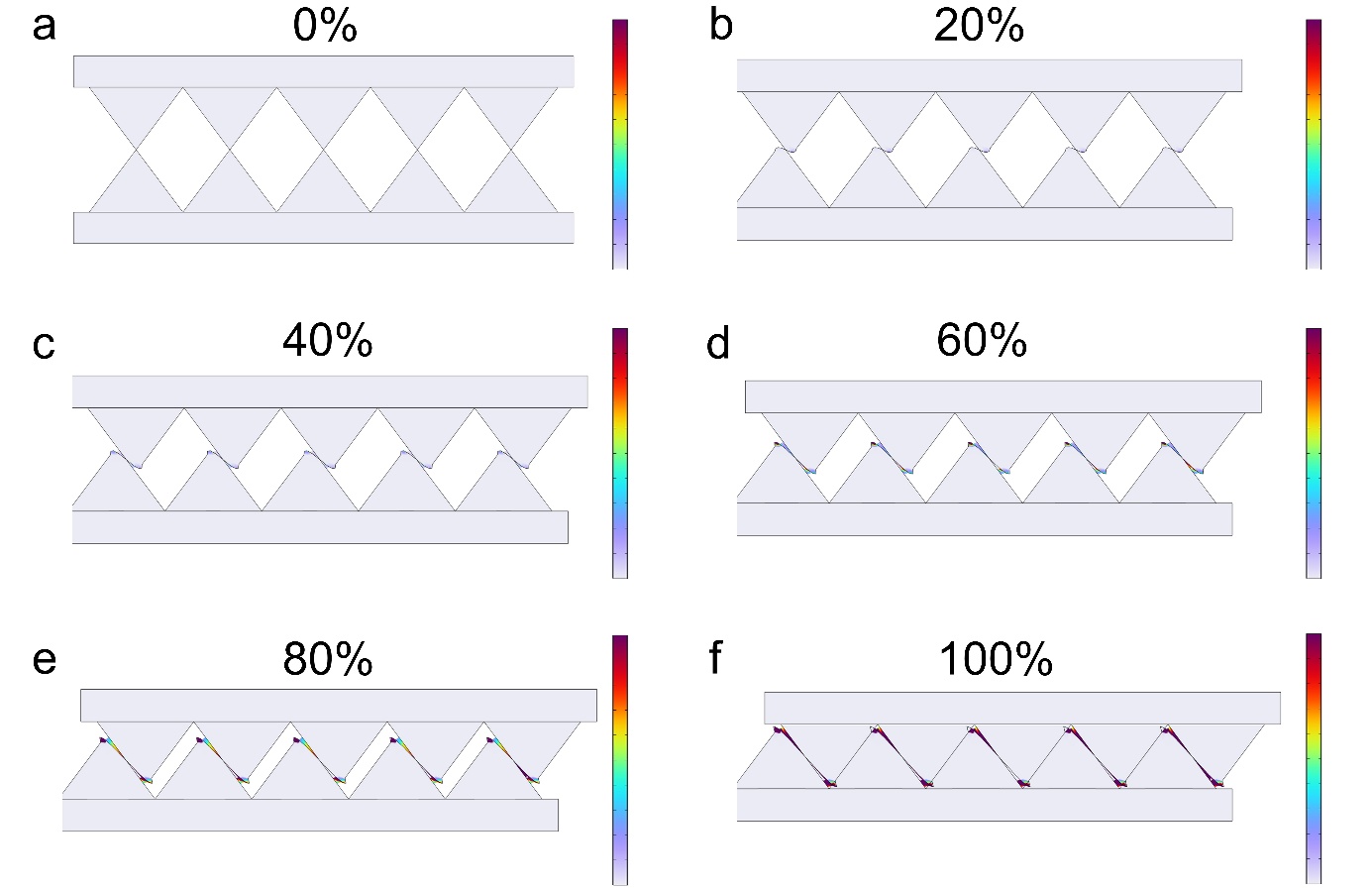


**Fig. S12 Von mise stress distribution maps of the pyramid-stacked structure as a function of displacement along the Y-axis. a** 0%. **b** 20%. **c** 40%. **d** 60%. **e** 80%. **f** 100%.


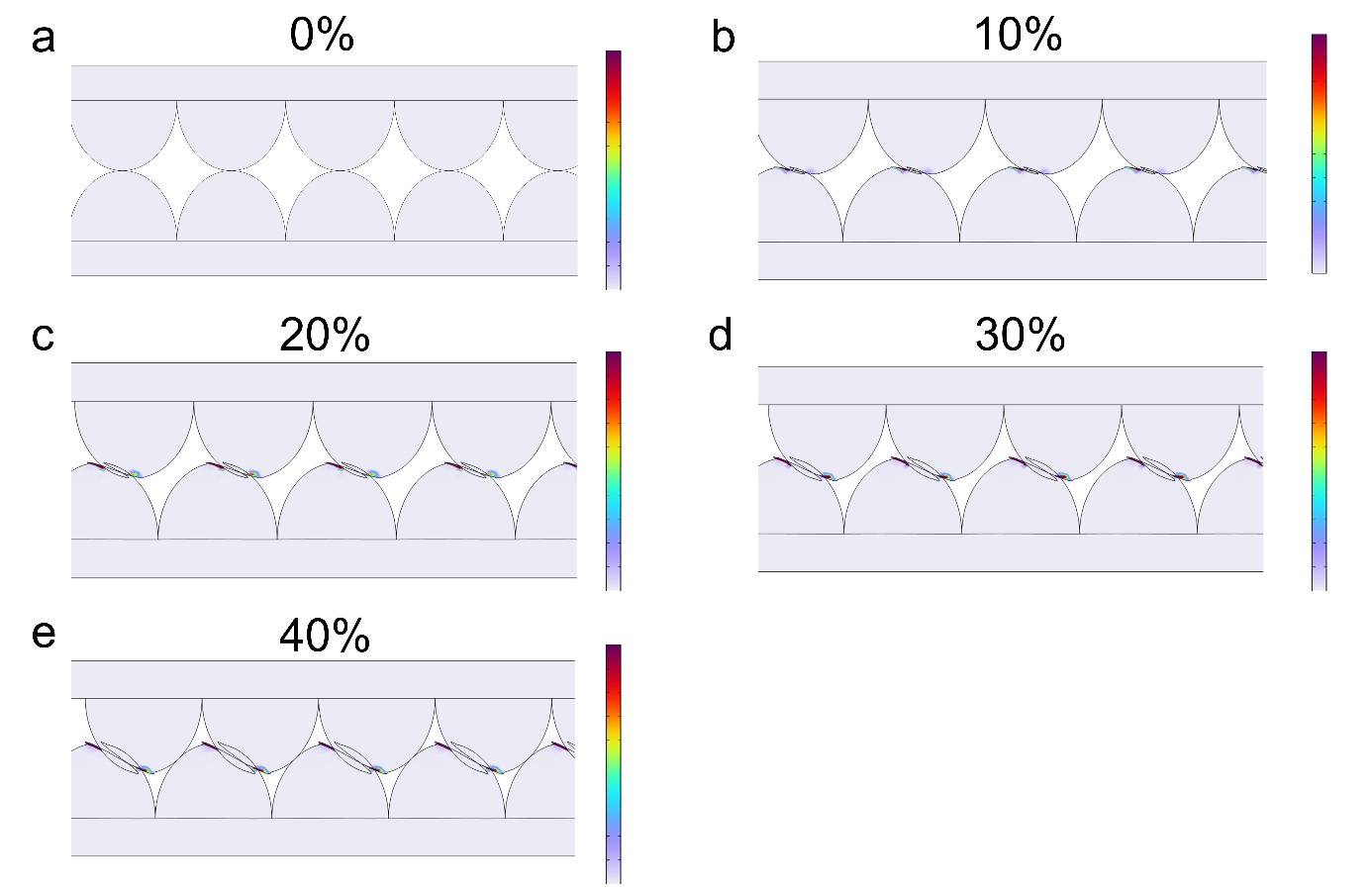


**Fig. S13 Von mise stress distribution maps of the island-stacked structure as a function of displacement along the Y-axis. a** 0%. **b** 10%. **c** 20%. **d** 30%. **e** 40%.


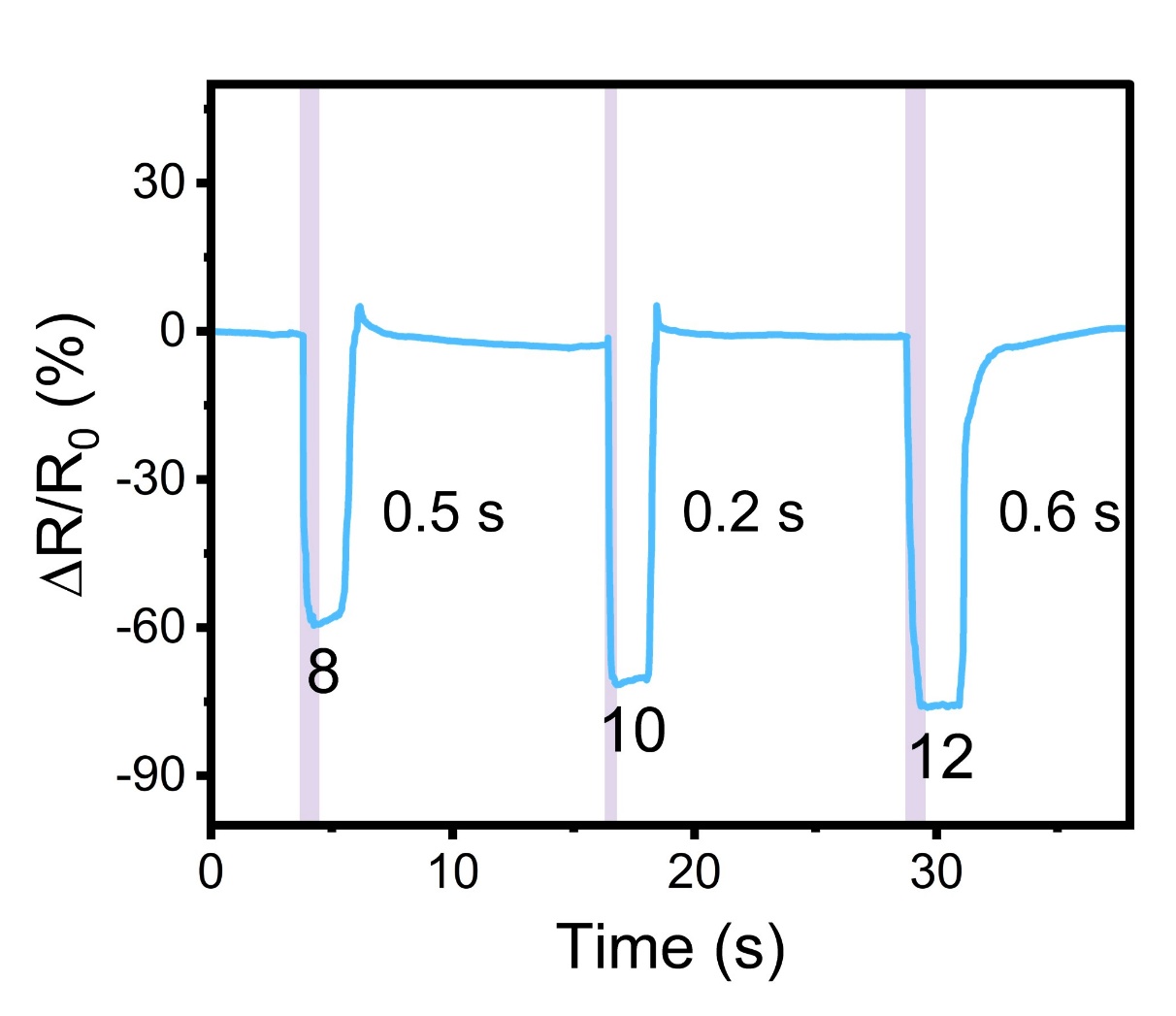


**Fig. S14 comparison of the resistance changes and response times of stacked devices with different layer numbers under the same pressure stimulus.**

**
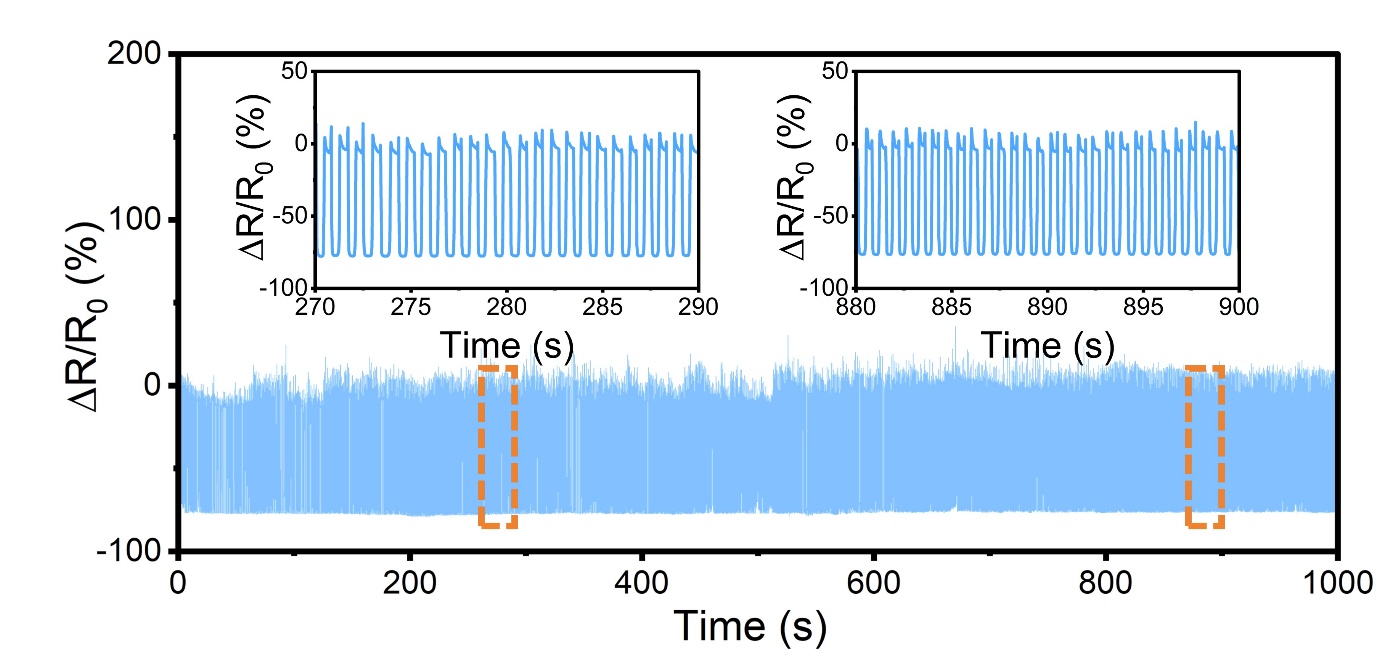
**

**Fig. S15 Long-term stability of the sensor device in a simulated seawater saline environment (NaCl solution).**

**
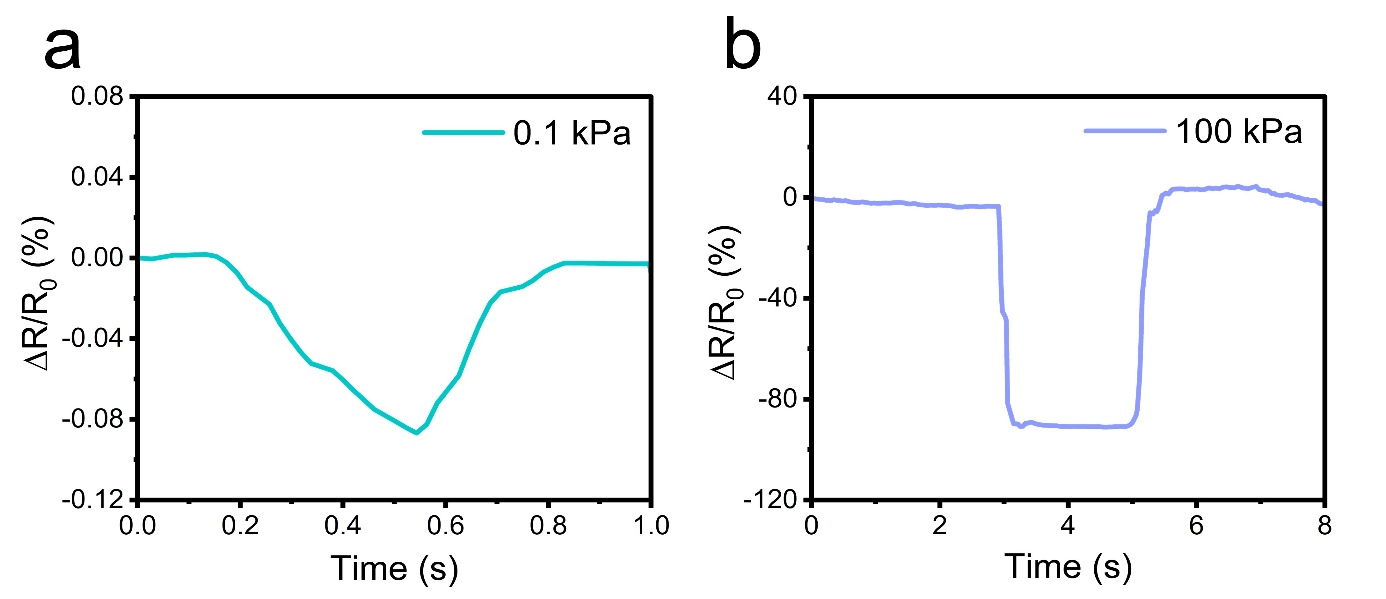
**

**Fig. S16 Detection limit tests of the device for temperature and pressure sensing. a** P = 0.1 kPa. **b** P = 100 kPa.


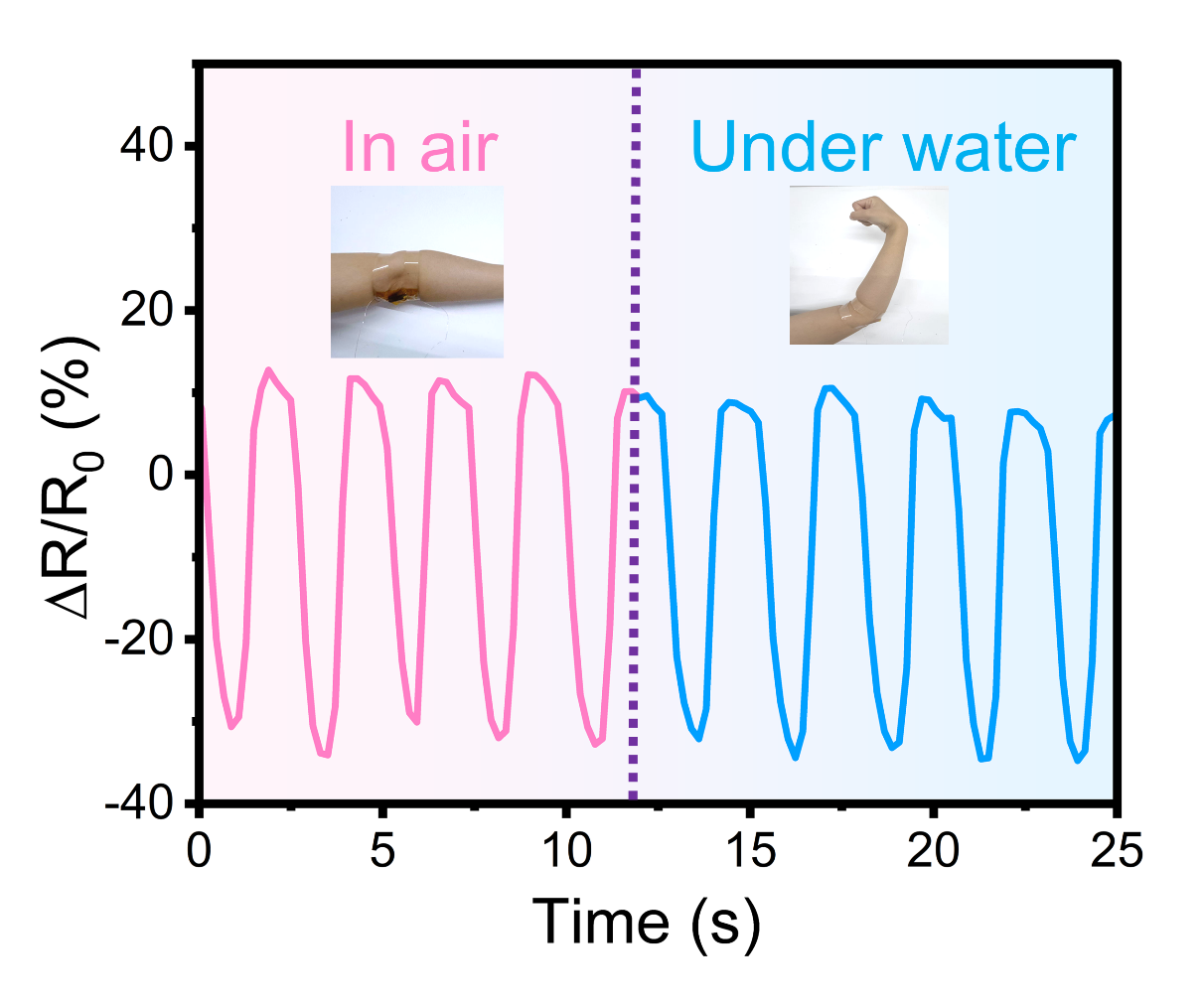


**Fig. S17 Pressure sensing response of the Bi_2_Se_3_/CP sensor during repeated elbow flexion, demonstrating its operational stability for human motion monitoring.**


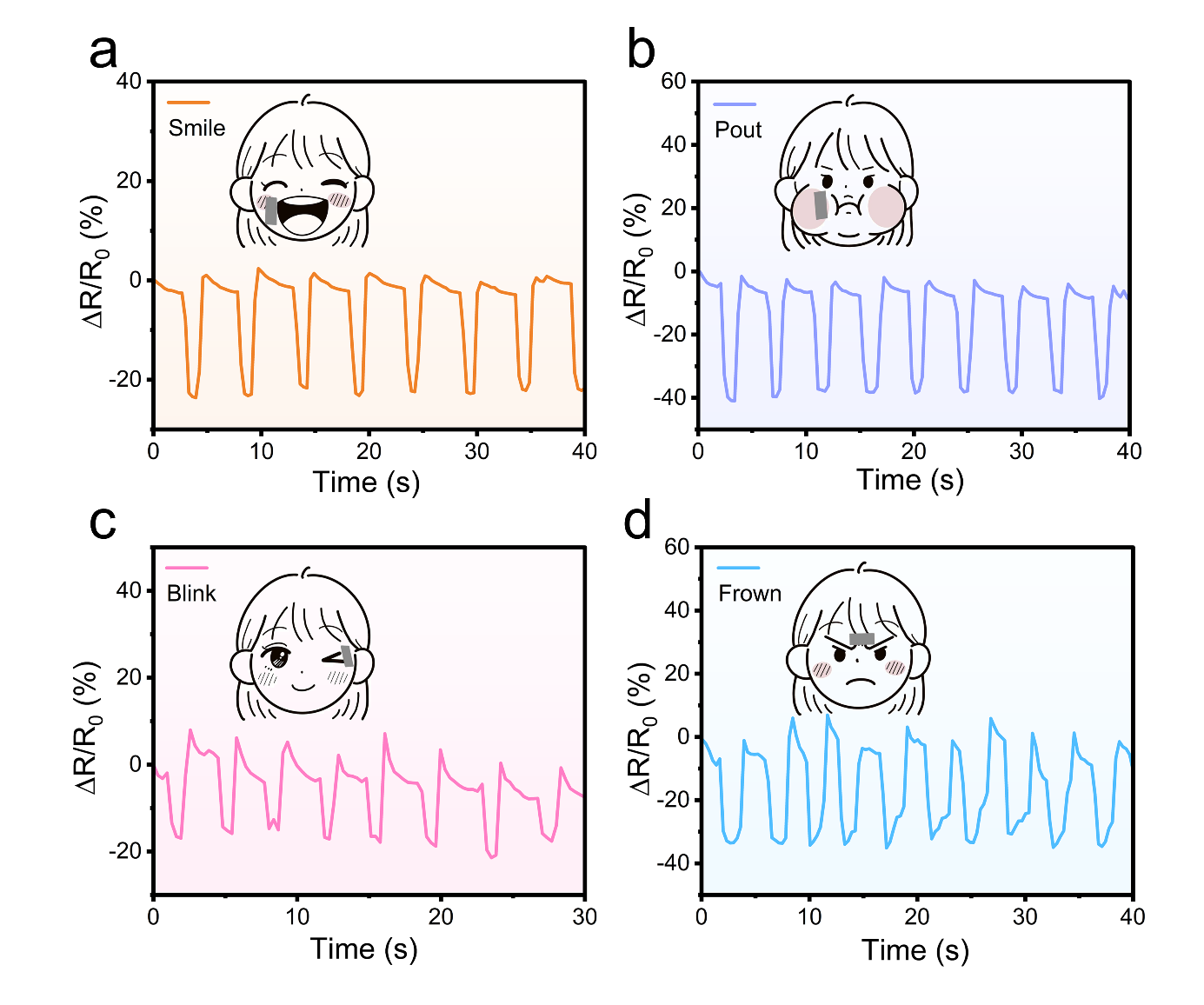


**Fig. S18 Detection of facial microexpressions using a Bi_2_Se_3_/CP sensors mounted on moist skin, simulating post-exercise or underwater conditions. a** Smile, **b** Pout, **c** Blink, and (**d**) Frown.The sensor reliably captures distinct signal patterns corresponding to each expression, demonstrating its potential for high-precision microexpression recognition in environments involving sweat or water exposure. This capability supports applications in post-exercise health monitoring, psychological analysis, emotion recognition, and wearable sensing under diverse conditions.


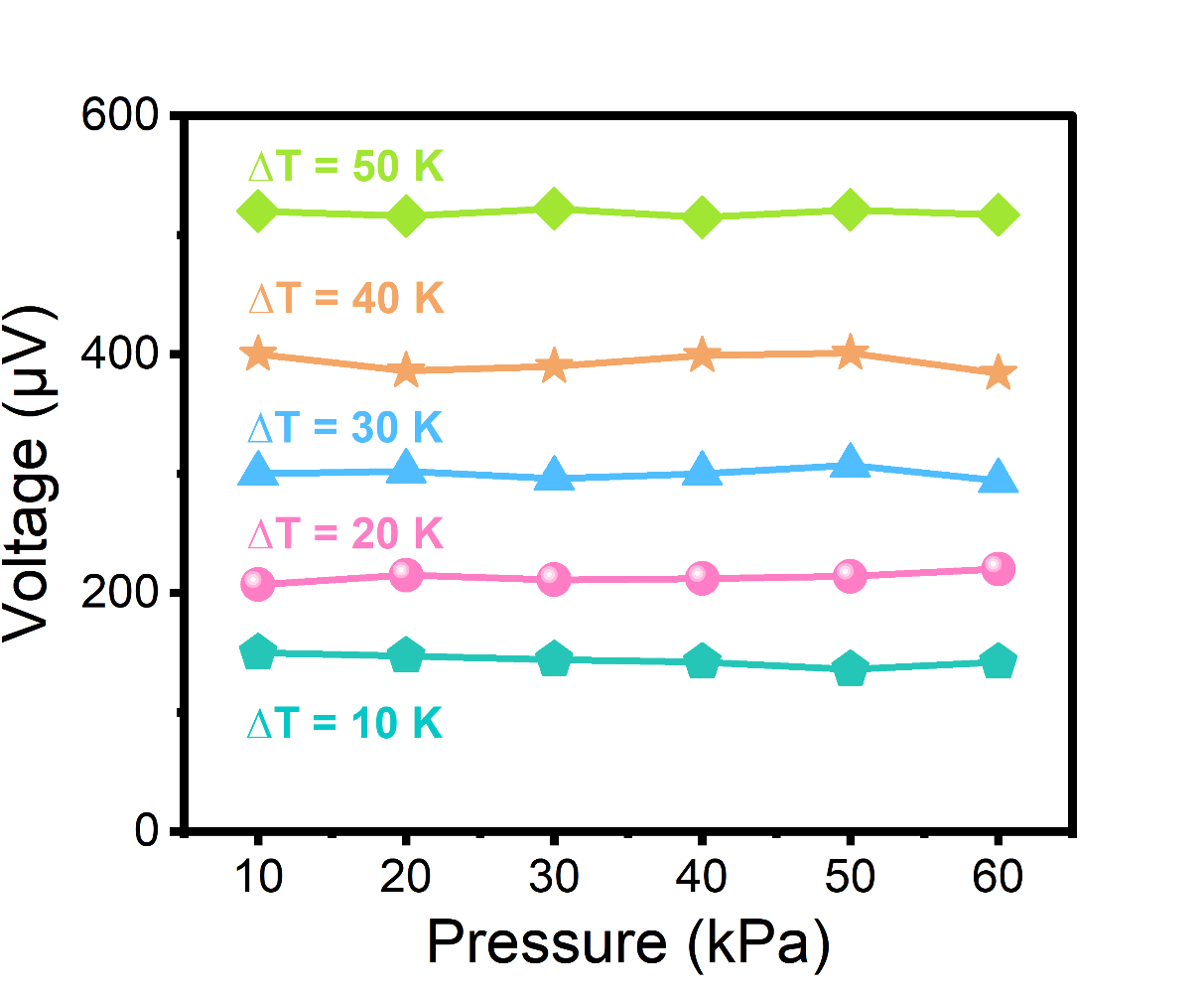


**Fig. S19 Stability evaluation of a 10-layer Bi_2_Se_3_/CP film stacked dual-mode sensor under varying pressure conditions.**

The output voltage was measured at different temperature differences (Δ*T*) while progressively increasing the applied pressure. Δ*T* represents the temperature difference between the thermostatically controlled end and the test end. The results demonstrate that the temperature sensing capability remains stable and unaffected by pressure variations.


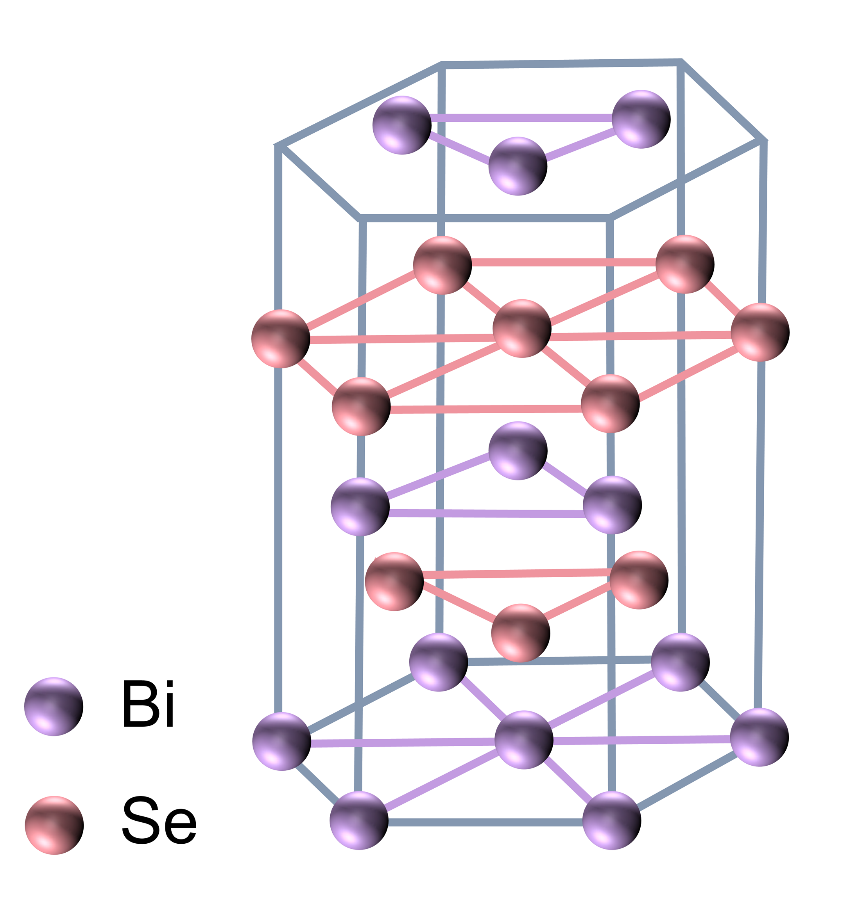


**Fig. S20 Layered crystal structure of Bi_2_Se_3_.**

The schematic illustrates the characteristic quintuple-layer arrangement (Se–Bi–Se–Se–Bi–Se) with weak van der Waals interactions between adjacent layers.


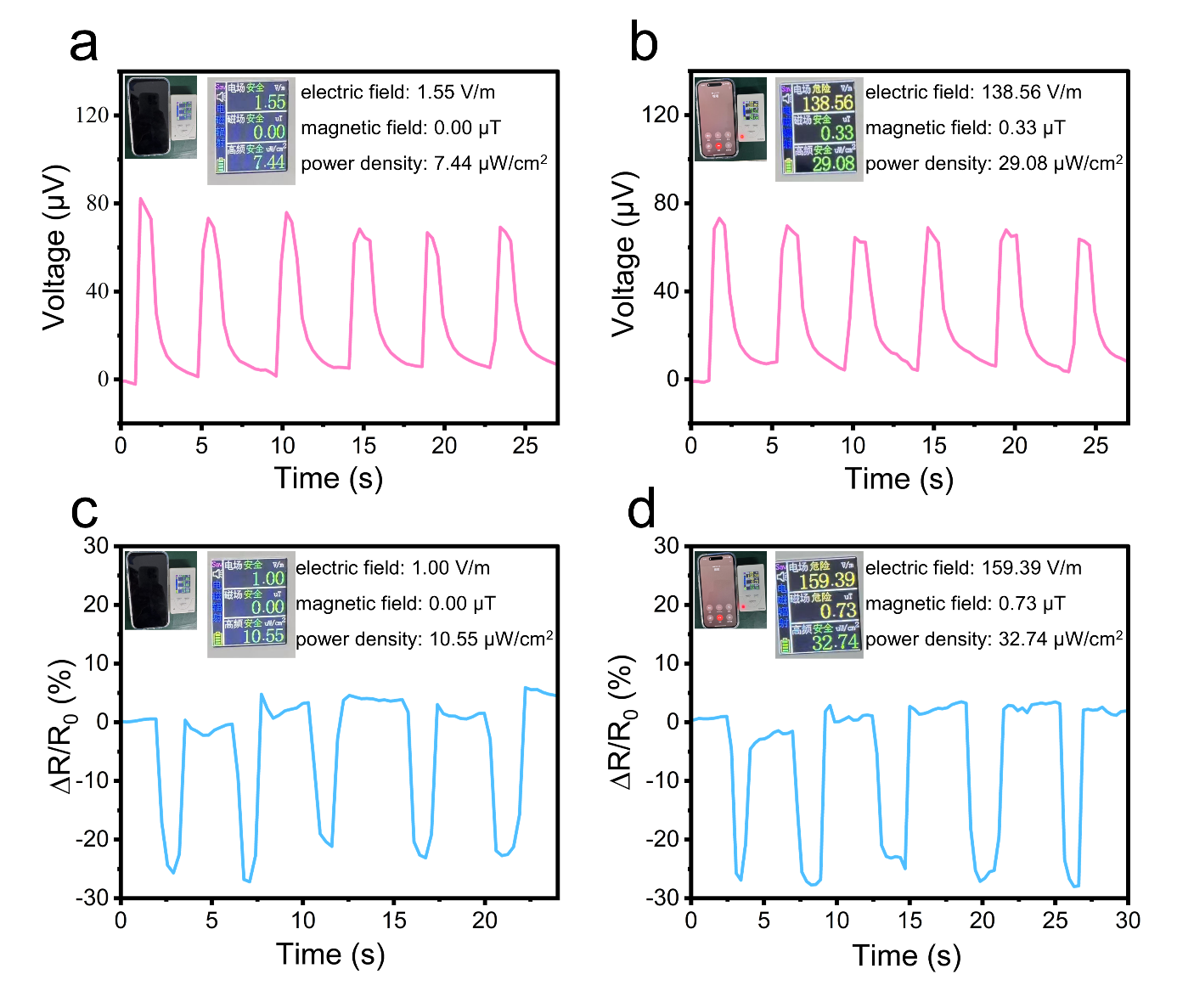


**Fig. S21 Voltage and resistance signal outputs of the dual‑mode sensor under different electromagnetic field conditions in air. a, b** Voltage output of the temperature sensor when placed near a phone in standby mode (a) and calling mode (b). Resistance output under under the same electromagnetic field conditions: (**c**) standby mode and (**d**) calling mode.


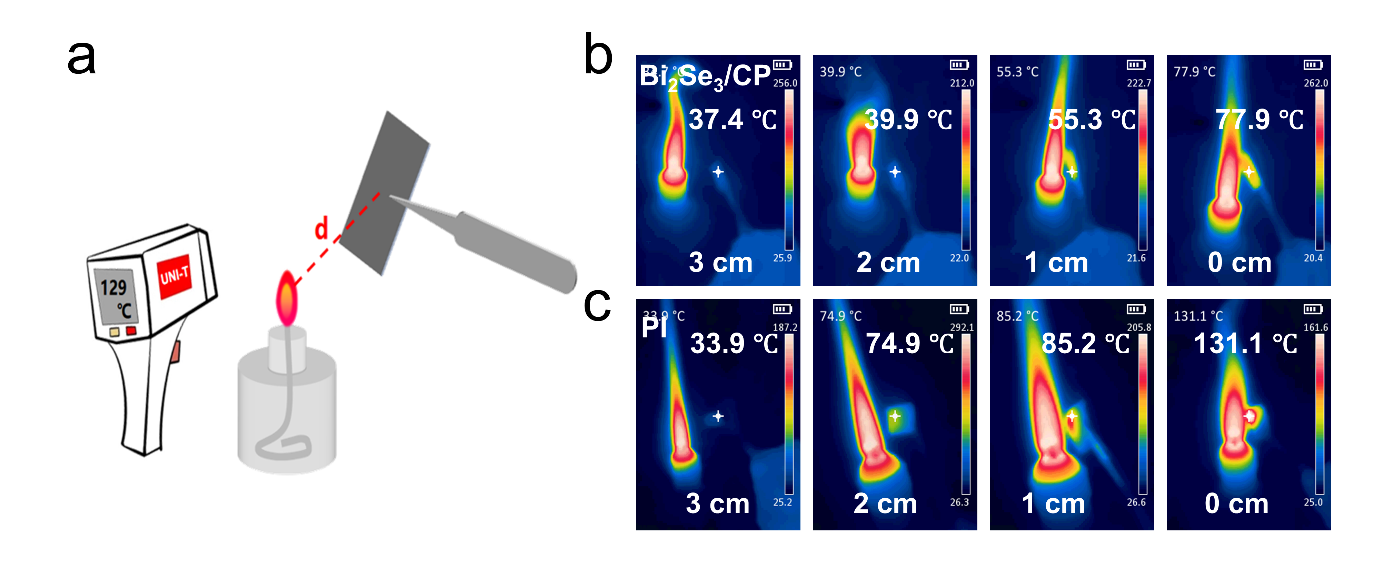


**Fig. S22 Thermal response of Bi_2_Se_3_/CP and PI films during gradual approaching to an alcohol lamp flames. a** Schematic of the experimental setup, showing the Bi_2_Se_3_/CP composite film positioned near the outer flame of an alcohol lamp, as visualized by an infrared camera. **b** Temperature profile of the Bi_2_Se_3_/CP composite film recorded by the infrared camera as the distance to the flame decreases from 3 cm to 0 cm. **c** Corresponding temperature profile of a polyimide (PI) film under identical conditions.


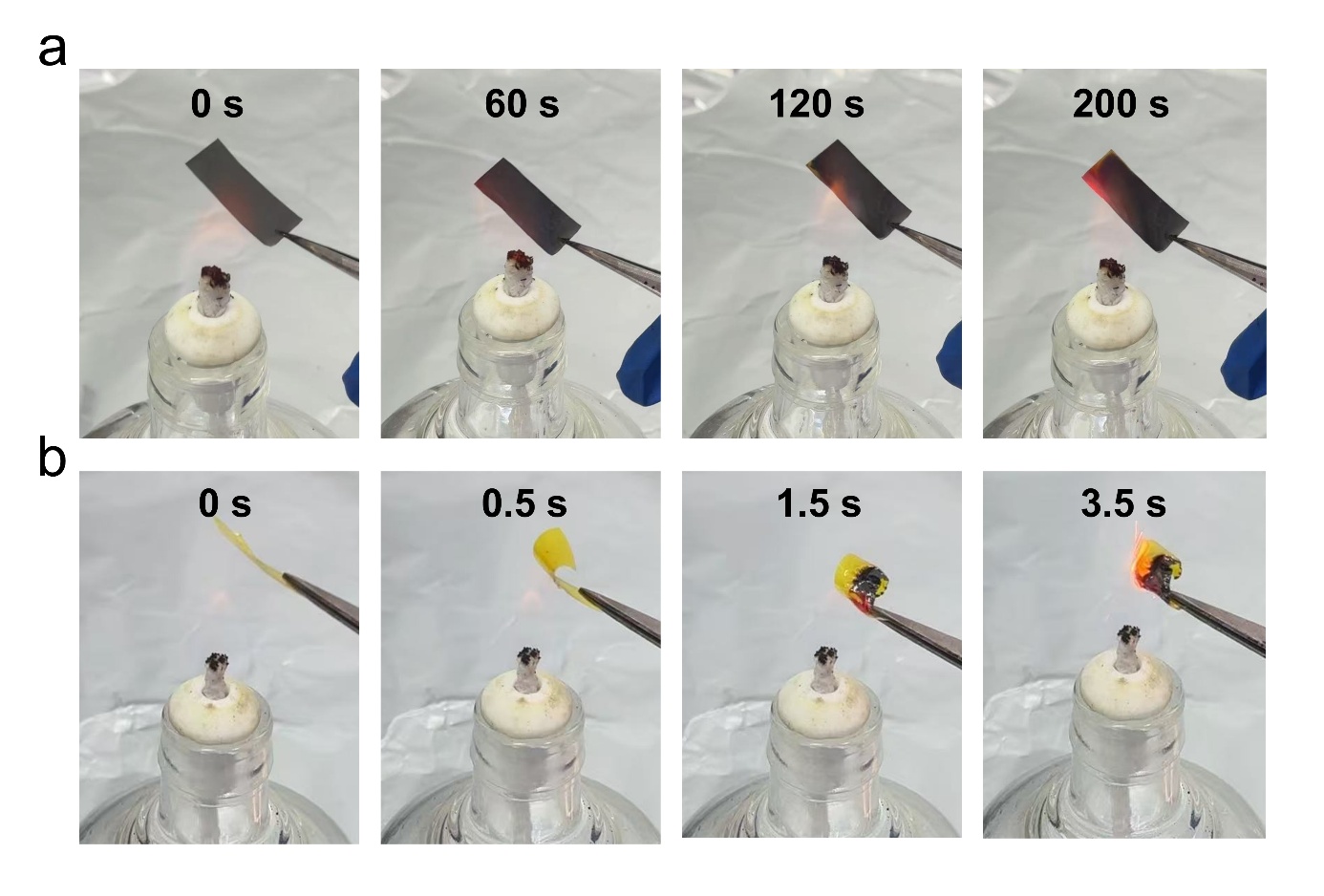


**Fig. S23 Ignition and combustion behavior of Bi_2_Se_3_/CP and PI films upon exposure to an alcohol lamp flame. a** The Bi_2_Se_3_/CP composite film remained unignited after 200 s of flame exposure, exhibiting no noticeable deformation, flame formation, or structural damage. **b** In contrast, the PI film bent and deformed immediately upon flame contact, with visible charring observed at 1.5 s and ignition occurring at 3.5 s.


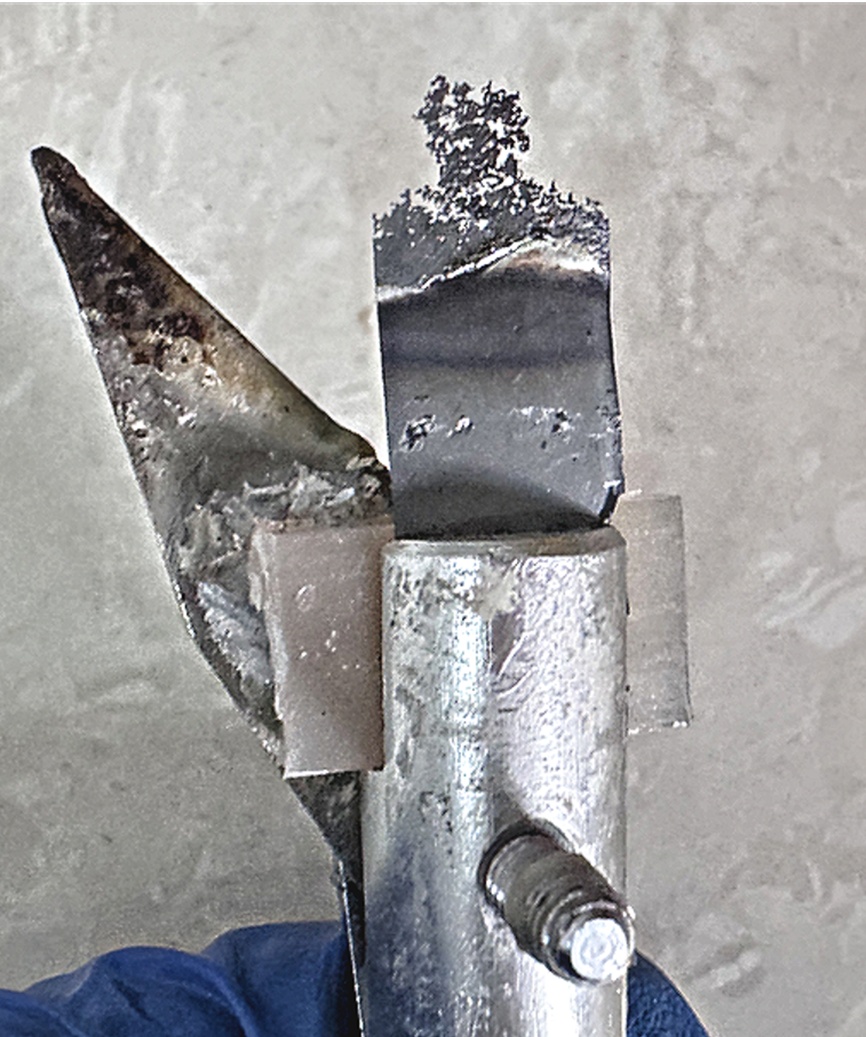


**Fig. S24 Combustion behavior of the Bi_2_Se_3_/CP composite film evaluated by oxygen index testing under controlled oxygen-to-nitrogen flow ratio.**

At oxygen concentration below 50%, the film did not ignite after 30 s of continuous flame exposure, and no visible surface changes were observed. At an oxygen concentration of 50%, the film exhibited partial decomposition after 30 s of exposure but failed to sustain ignition; decomposition ceased immediately upon removal of the flame.


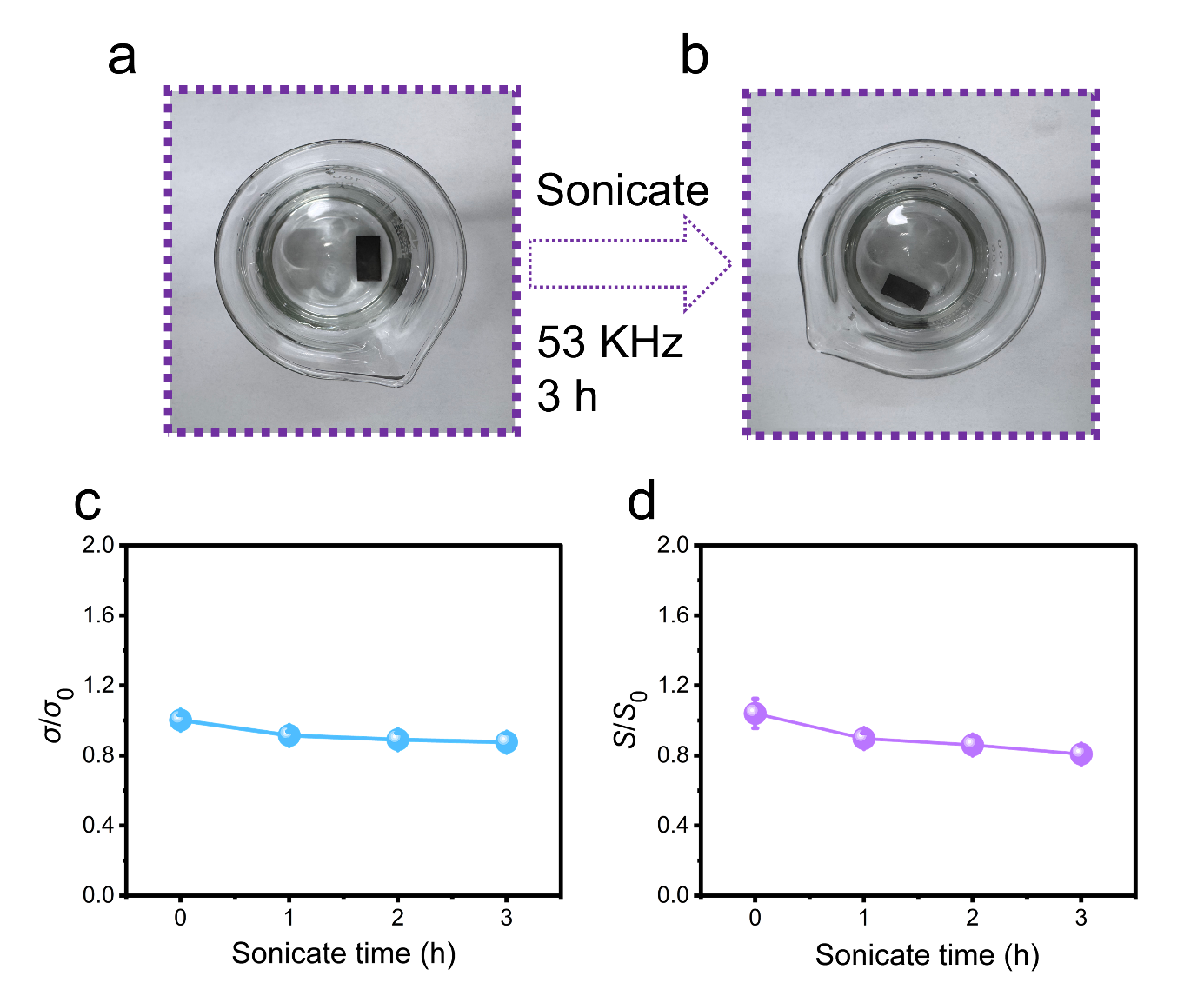


**Fig. S25 Mechanical stability of Bi_2_Se_3_/CP composite film under ultrasonication. a, b** Photographs of the film deposited at −0.02 V before (a) and after (b) ultrasonication at 53 KH_Z_ for 3h, showing no visible detachment or sedimentation. **c, d** Corresponding changes inelectrical conductivity (c) and Seebeck coefficient (d) before and after ultrasonication, demonstrating minimal degradation and robust mechanical integrity.


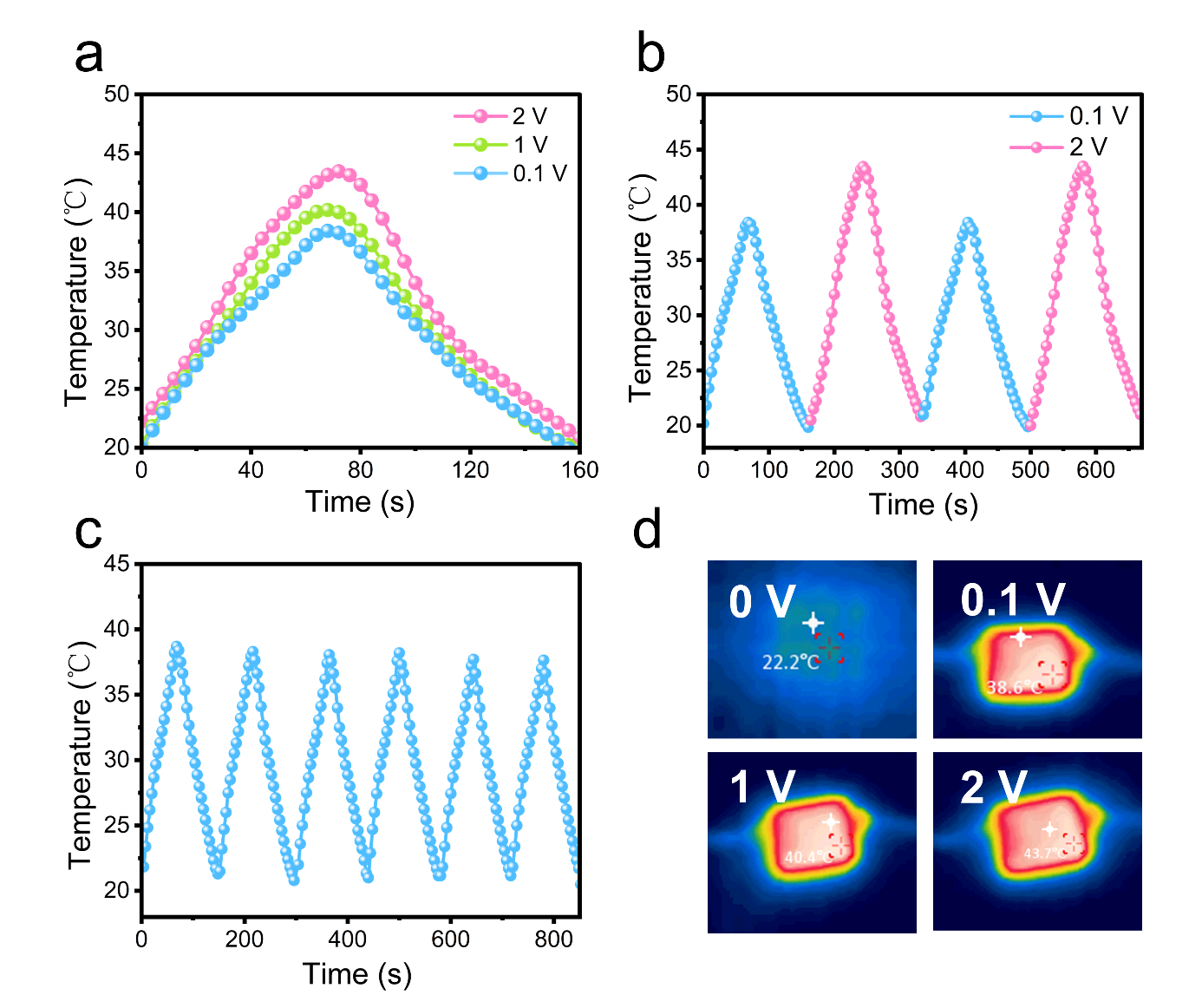


**Fig. S26 Joule heating performance of a ten‑layer Bi_2_Se_3_/CP stacked sensor. a** Time‑dependent temperature profiles under different applied voltages. **b** Temperature response to alternating voltages between 0.1 and 2 V. **c** Cyclic performance test at 0.1 V, demonstrating stable and reversible Joule heating behavior. **d** Infrared thermal images of the device at various applied voltages.

**Table S1 Abbreviations used in this paper and their corresponding physical units**

| NO. | Symbols/acronyms | Full names | Units |
| --- | --- | --- | --- |
| 1 | *n* | Carrier concentration | cm^−3^ |
| 2 | *μ* | Carrier mobility | cm^2^ V^−1^ s^−1^ |
| 3 | *σ* | Electrical conductivity | S cm^−1^ |
| 4 | *S* | Seebeck coefficient | μV K^−1^ |
| 5 | *PF* | Power factor | μW m^−1^ K^−2^ |
| 6 | U | Output voltage | mV |
| 7 | Δ*R*/*R*_0_ | Change in resistance / resistance at initial state | - |
| 8 | E*_f_* | Fermi level | eV |
| 9 | *Φ* | Work function |  |
| 10 | Δ*T* | Temperature gradients |  |
| 11 | EMI | Electromagnetic interference | dB |
| 12 | EMI SE | Electromagnetic shielding effectiveness |  |
| 13 | SE_R_ | Reflection loss |  |
| 14 | SE_A_ | Absorption loss |  |
| 15 | SE_M_ | Multiple reflections loss |  |
| 16 | SE_T_ | Total Shielding Effectiveness |  |
| 17 | *δ* | Skin depth | μm |
| 18 | T | Respiratory cycle | s |
| 19 | RR | Respiratory rate | bpm |
| 20 | CP | Carbon paper | - |
| 21 | PI | Polyimide | - |
| 22 | TEG | Thermoelectric generator | - |
| 23 | LSV | Linear sweep voltammetry | - |
| 24 | XRD | X-ray diffractometer | - |
| 25 | SEM | Scanning electron microscope | - |
| 26 | EDS | Energy dispersive X-ray spectrometer | - |
| 27 | XPS | X-ray photoelectron spectroscopy | - |
| 28 | *r*_b_ | Bending radius | mm |
| 29 | ƒ_FOM_ | Flexibility figure of merit | - |
| 30 | *S/S*_0_ | Seebeck coefficient / seebeck coefficient at initial state | - |
| 31 | WE | Working electrode | - |
| 32 | CE | Counter electrode | - |
| 33 | RE | Reference electrode | - |
| 34 | SCE | Saturated calomel electrode | - |
| 35 | DFT | Density functional theory | - |
| 36 | VASP | Vienna Ab initio Simulation Package | - |
| 37 | PAW | Projector-augmented wave | - |
| 38 | GGA | Generalized gradient approximation | - |
| 39 | PBE | Perdew–Burke–Ernzerhof | - |

**Table S2 Comparison of thermoelectric properties between Bi_2_Se_3_/CP films deposited at different potentials and original CP**

| Samples | Deposition potential (V) | *S* (μV K^−1^) | *σ* (S cm^−1^) | *μ* (cm^2^ V^−1^ s^−1^) | *n* (×10^20^ cm^−3^) | *PF* (μW m^−1^ K^−2^) |
| --- | --- | --- | --- | --- | --- | --- |
| CP | / | 6.9 | 2505.0 | 20.2 | 7.7 | 11.9 |
| Bi_2_Se_3_/CP | −0.06 | −30.1 | 562.3 | 7.9 | 4.2 | 51.7 |
| Bi_2_Se_3_/CP | −0.04 | −34.3 | 656.3 | 14.3 | 2.6 | 77.2 |
| Bi_2_Se_3_/CP | −0.02 | −36.9 | 779.3 | 19.7 | 2.5 | 106.0 |
| Bi_2_Se_3_/CP | 0.00 | −33.7 | 590.0 | 6.1 | 5.6 | 67.0 |

**Table S3 Response and sensitivity comparison of different temperature/pressure sensors**

| Material | Underwater operation | Temperature response time (s) | Temperature sensitivity (μV K^–1^) | Pressure response time (s) | Pressure sensitivity and limit | Ref. |
| --- | --- | --- | --- | --- | --- | --- |
| PEDOT:PSS/PU | No | < 2 | 32.8 | < 0.02 | 4.3 kPa^–1^ (0.1-3 kPa) | [S^[[1]](#endnote-1)^] |
|  |  |  |  |  | 28.9 kPa^–1^ (3-20 kPa) |  |
| Bi_2_Te_3_ | No | 0.95 | 426.4 | 0.3 | 0.12 kPa⁻¹ (0-10 kPa) | [S^[[2]](#endnote-2)^] |
| PTTS | No | 0.05 | 21.5 | - | 0.3488 kPa^–1^ (0-110 kPa) | [S^[[3]](#endnote-3)^] |
| PEDOT:PSS/CNTs | No | 17.97 | 25.7 | 0.831 | 0.0093 kPa^–1^ | [S^[[4]](#endnote-4)^] |
| p-PDMS/MWCNT | No | 1.44 | 5.57 | 0.082 | 0.09 kPa^–1^ (0-6.49 kPa) | [S^[[5]](#endnote-5)^] |
| Fully paper-integrated sensor | Yes | - | - | 0.214 | 12.6 kPa^–1^ (0-0.6 kPa) | [S^[[6]](#endnote-6)^] |
|  |  |  |  |  | 4.3 kPa^–1^ (0.6-60.4 kPa) |  |
| Porous TPU | Yes | - | - | 0.19 | 0.62 kPa^–1^ (50-600 kPa) | [S^[[7]](#endnote-7)^] |
| MXene/PEDOT:PSS/WPU | Yes | 1.31 | 6.33 | - | - | [S^[[8]](#endnote-8)^] |
| AKD-GNPs/CNFs/CPaper | Yes | - | - | 0.3 | 0.019 kPa^–1^ (50-316.5 kPa) | [S^[[9]](#endnote-9)^] |
| Bi_2_Se_3_/CP | Yes | 0.95 | 9.8 | 0.2 | 0.0094 kPa^–1^ (0.1-100 kPa) | This  work |

Abbreviations:

PEDOT:PSS/PU: Poly(3,4-ethylenedioxythiophene):poly(styrenesulfonate)/polyurethane composite;

PTTS: Porous thermoelectric temperature sensor;

PEDOT:PSS/CNTs: Poly(3,4-ethylenedioxythiophene):poly(styrenesulfonate)/carbon nanotubes composite;

p-PDMS/ MWCNT: Porous polydimethylsiloxane/multi-walled carbon nanotube composite;

Porous TPU: Porous thermoplastic polyurethane;

MXene/PEDOT:PSS/WPU: MXene/poly(3,4-ethylenedioxythiophene):poly(styrenesulfonate)/waterborne polyurethane composite;

AKD-GNPs/CNFs/CPaper: Alkyl ketene dimer-coated graphene nanoplatelets/cellulose nanofibers/cellulose paper composite;

**Supplementary References**

1. F. Zhang, Y. Zang, D. Huang, C. Di, D. Zhu et al., Flexible and self-powered temperature-pressure dual-parameter sensors using microstructure-frame-supported organic thermoelectric materials. Nat. Commun. **6**, 8356 (2015). <https://doi.org/10.1038/ncomms9356> [↑](#endnote-ref-1)
2. H. Yu, Z. Hu, J. He, Y. Ran, Y. Zhao et al., Flexible temperature-pressure dual sensor based on 3D spiral thermoelectric Bi_2_Te_3_ films. Nat. Commun. **15**, 2521 (2024). <https://doi.org/10.1038/s41467-024-46836-1> [↑](#endnote-ref-2)
3. Q. Hong, T. Liu, X. Guo, Z. Yan, W. Li et al., 3D dual-mode tactile sensor with decoupled temperature and pressure sensing: toward biological skins for wearable devices and smart robotics. Sens. Actuators B Chem. **404**, 135255 (2024). <https://doi.org/10.1016/j.snb.2023.135255> [↑](#endnote-ref-3)
4. Q. Mou, B. Qu, C. Wang, L. Zeng, C. Liu et al., A deep learning-assisted interference-free and real-time decoupling multimodal sensor for tactile sensing. Chem. Eng. J. **526**, 171385 (2025). <https://doi.org/10.1016/j.cej.2025.171385> [↑](#endnote-ref-4)
5. X. Han, X. Wen, Y. Deng, X. Zhang, X. Dong, et al., Crosstalk-suppressed flexible sensor for simultaneous pressure-temperature detection. Sens. Actuators A Phys. **399**, 117450 (2026). <https://doi.org/10.1016/j.sna.2026.117450> [↑](#endnote-ref-5)
6. Y. Wei, X. Shi, Z. Yao, J. Zhi, L. Hu et al., Fully paper-integrated hydrophobic and air permeable piezoresistive sensors for high-humidity and underwater wearable motion monitoring. npj Flex. Electron. **7**, 13 (2023). <https://doi.org/10.1038/s41528-023-00244-5> [↑](#endnote-ref-6)
7. Q. Wang, X. Fu, H. Xu, J. Fan, Y. Zou et al., Self-powered underwater pressing and position sensing and autonomous object grasping with a porous thermoplastic polyurethane film sensor. Adv. Funct. Mater. **34**, 2315648 (2024). <https://doi.org/10.1002/adfm.202315648> [↑](#endnote-ref-7)
8. Z. Yang, J. Li, K. Liao, Q. Zeng, X. Zhang et al., A flexible, self-adhesive, and waterproof Mxene-based composite thermoelectric film with a brick-and-mortar structure for advanced temperature sensing and multifunctional applications. Adv. Funct. Mater. **35**, 2525831 (2025). <https://doi.org/10.1002/adfm.202525831> [↑](#endnote-ref-8)
9. H. Liu, W. Wang, H. Xiang, H. Wu, Z. Li et al., Paper-based flexible strain and pressure sensor with enhanced mechanical strength and super-hydrophobicity that can work under water. J. Mater. Chem. C **10**, 3908−3918 (2022). <https://doi.org/10.1039/D1TC04697G> [↑](#endnote-ref-9)
